# Biologic therapy is associated with reduced ocular disease in psoriasis: a real-world study

**DOI:** 10.1038/s41433-026-04274-x

**Published:** 2026-02-05

**Authors:** Shoham Kubovsky, Natan Lishinsky-Fischer, Itay Chowers, Yuval Ramot, Jaime Levy

**Affiliations:** 1 Ophthalmology Department, Hadassah Medical Center, Jerusalem, Israel; 2 Faculty of Medicine, The Hebrew University of Jerusalem, Jerusalem, Israel; 3 Department of Dermatology, Hadassah Medical Center, Jerusalem, Israel

**Keywords:** Outcomes research, Risk factors

## Abstract

**Background/objectives:**

Psoriasis is a systemic immune-mediated disease with ocular involvement. While biologic therapies reduce cardiovascular and musculoskeletal comorbidities, their impact on ocular health is not well characterised. We aimed to assess whether biologic therapy is associated with reduced ocular disease risk in psoriasis patients.

**Subjects/methods:**

We performed a large-scale, retrospective cohort study using the TriNetX Global Collaborative Network (>160 million patients worldwide). Adults with psoriasis initiating biologic therapy were compared with those receiving non-biologic systemic treatments. Cohorts were matched 1:1 by propensity scoring for demographic and clinical variables. Sixty-eight ocular outcomes were assessed over 6 to 120 months. Hazard ratios (HRs) were estimated using proportional hazards models.

**Results:**

Among 30,911 biologic-treated and 35,832 non-biologic-treated patients with psoriasis, biologic therapy was consistently associated with reduced risk of ocular surface and corneal inflammation. The strongest associations were seen for dry eye disease (mean HR = 0.55, 95% confidence interval [CI; 0.42, 0.66], *p* = 0.0007), conjunctivitis (mean HR = 0.71, 95% CI [0.59, 0.86], *p* = 0.01), and keratitis (mean HR = 0.40, 95% CI [0.3, 0.56], *p* = 0.0007). These lower-risk associations were evident from 6 months and remained observable in the 10-year analysis window. No consistent reduction was observed for retinal or vitreous disease.

**Conclusions:**

Biologic therapy in psoriasis was associated with a lower risk of ocular surface disease. These observational findings may inform interdisciplinary management and consideration of ocular outcomes in treatment decisions.

## Introduction

Psoriasis, a chronic immune-mediated inflammatory skin disease, is increasingly recognised as a systemic disorder with potential ocular involvement. Ocular manifestations in psoriasis without arthritis are varied, affecting multiple structures such as the eyelids, conjunctiva, and cornea, with prevalence estimates across studies ranging from ~10% up to >80% depending on study design and patient population. Dry eye disease, blepharitis, and conjunctivitis are among the most commonly reported ocular conditions in psoriasis [1–3]. In contrast, patients with psoriatic arthritis (PsA) appear to carry a substantially higher risk of intraocular inflammation, particularly uveitis. Meta-analytic data indicate that PsA is more strongly associated with uveitis than skin-limited psoriasis. Moreover, clinical studies show that PsA-associated uveitis tends to have more frequent anterior segment complications (e.g., cataract, ocular hypertension) compared with uveitis in psoriasis alone [4–6]. Immunopathogenesis is thought to involve overexpression of IL-17, IL-23, and TNF-α, which promote epithelial disruption, neutrophil infiltration, and chronic inflammatory damage to ocular tissues [7, 8].

Biologic therapies targeting these cytokines have transformed the management of psoriasis. Tumor necrosis factor inhibitors, IL-12/23 inhibitors, IL-17 inhibitors, and IL-23 inhibitors improve cutaneous and joint outcomes while reducing systemic inflammation. Given the overlap between immune pathways implicated in psoriasis and ocular inflammatory disease, it has been hypothesised that biologic therapies may be associated with differences in ocular outcomes. However, population-level evidence regarding such associations remains limited.

In this study, we leveraged the TriNetX Global Collaborative Network, a large federated electronic health record database, to evaluate whether biologic therapy in psoriasis is associated with a reduced risk of ocular disease compared with non-biologic systemic treatments. We examined outcomes across the ocular surface, cornea, anterior chamber, retina, and optic nerve over both short- and long-term follow-up. We hypothesised that biologic therapy would be associated with a lower incidence of ocular surface and anterior segment inflammation, acknowledging that any identified differences would represent observational associations and not causal effects or therapeutic benefits.

## Methods

TriNetX is a comprehensive database integrating electronic medical records (EMRs) from multiple healthcare organisations (HCOs). All EMRs are encoded using the International Classification of Diseases, 10th Revision (ICD-10). For this study, we utilised the largest TriNetX database, the “Global Collaborative Network,” which, as of April 2025, contained data from 165,981,746 patients across 146 healthcare organisations in 18 countries, with records dating back to January 2006. TriNetX complies with the Health Insurance Portability and Accountability Act (HIPAA), a U.S. federal law ensuring the privacy and security of healthcare data. Additionally, it is certified under the ISO 27001:2013 standard and maintains an Information Security Management System (ISMS) for data protection. Consequently, informed consent was not required for this study.

### Inclusion criteria

We identified two groups of patients using the built-in query function in TriNetX. The first group included patients with confirmed diagnosis of psoriasis who were prescribed any available biologic treatment from the following classes: TNF inhibitors, IL-12/23 inhibitors, IL-17A inhibitors, IL-17A/F inhibitors, and IL-23 inhibitors. The second group consisted of patients with a confirmed diagnosis of psoriasis who were prescribed any of the following systemic treatments: Methotrexate, Cyclosporine, Acitretin, Apremilast, or Dimethyl Fumarate. Patients in both groups were included only if the treatment was initiated after the initial diagnosis of psoriasis. A complete list of drug names and corresponding ICD-10 codes is provided in Supplementary Table S1.

For each cohort, the index event was defined as the first recorded prescription for any of the enlisted medications. 68 ocular diagnoses were chosen as outcome measurements (Supplementary Table S2). These included common ocular diagnoses known to be related to psoriasis based on previous literature [2] and diagnoses that have been hypothesised to share similar inflammatory pathways. These diagnoses were categorised into three categories: (1) external eye diseases, (2) retinal and vitreous diseases and (3) cataract and other ocular-related diagnoses. Inguinal hernia (ICD-10 code K40), acute appendicitis (K35), cholelithiasis (K80), dental caries (K02) and fracture at wrist and hand level (S62) were chosen to serve as negative control outcome.

### Exclusion criteria

Patients with no follow-up or a follow-up period of less than six months were excluded from the study. Additionally, patients with any record of systemic treatment for psoriasis were excluded from the biological treatment group, and those with any record of biological treatment were excluded from the systemic treatment group.

### Propensity score matching

One of TriNetX’s strengths is its built-in 1:1 propensity score matching (PSM) algorithm, which helps reduce, but cannot eliminate, confounding variables. In this analysis, PSM was applied to nine variables: Age at Index Date, number of females, number of patients identified as White, hypertensive diseases (I10-I1A), hyperlipidaemia (E78.5), diabetes mellitus (E08-E13), nicotine dependence (F17), long-term steroid use (Z79.52), and family history of ocular disorders (Z83.518). We present the basic characteristics of the cohorts both before and after PSM, and outcome measurements were compared in the matched cohorts.

### Statistical analysis

We set a follow-up time window of 6, 12, 24, 36, 48, 60 and 120 months after the index event. Our analysis included a survival approach, using Kaplan-Meier curves and log-rank test to estimate associations. Adjusted p-values were calculated for each outcome using Bonferroni correction. Hazard ratios (HRs) with 95% confidence intervals (95% CI) were calculated using a proportional-hazard model for 68 ocular outcomes and the negative control variable in both groups. HRs were computed on the TriNetX platform by conducting a series of tests using R’s Survival package (v3.2-3) and cross-checking the results with output from SAS (SAS Institute, Cary, NC, USA, version 9.4) to ensure validation. Proportionality tests were conducted to assess whether hazard rates remained constant over time. A *p* ≤ 0.01 was considered significant for nonproportionality.

### Sensitivity analysis

In addition to the primary analyses, we conducted a series of sensitivity analyses to assess the robustness of these associations, without inferring causality. These included stratifying patients by geographic network, expanding the set of PSM variables, incorporating several negative control variables, stratifying cohorts by psoriasis severity, and stratifying by biologic class.

## Results

A total of 30,911 and 35,832 patients were included in the biologic and non-biologic groups, respectively. Propensity score matching was performed separately for each follow-up period (6-120 months), ensuring balanced baseline characteristics (Table 1; Supplementary Tables S3–S8). Among the 68 ocular diagnoses analysed, 16 differed significantly in ≥3 follow-up periods, and 7 demonstrated consistent associations across all periods. Three of these were also clinically meaningful: conjunctivitis (mean HR 0.71, 95% CI [0.59, 0.86], *p* = 0.01), dry eye syndrome (mean HR 0.55, 95% CI [0.42,-0.66], *p* = 0.0007), and keratitis (mean HR 0.40, 95% CI [0.30,-0.56], *p* = 0.0007).

**Table 1:** Patient characteristics before and after propensity score matching for patients with confirmed diagnosis of psoriasis who were prescribed with biologic agents vs. patients with a confirmed diagnosis of psoriasis who were prescribed with non-biologic systemic therapy for a follow-up period of 6 months.

Characteristic Name	Before PSM				After PSM			
	Biological (n = 30,991)	Systemic (n = 35,832)	*P*	Std diff.	Biological (n = 25,278)	Systemic (n = 25,278)	*P*	Std diff.
Age at Index (mean ± SD)	47.71 ± 17.04	54.54 ± 17.42	**<0.0001**	**0.40**	51.04 ± 16.23	50.45 ± 17.0	<0.0001	0.04
White (%)	22,612 (73.11)	20,484 (59.38)	**<0.0001**	**0.29**	17,193 (68.02)	16,704 (66.08)	<0.0001	0.04
Female (%)	16,293 (52.68)	19,088 (55.33)	<0.0001	0.05	13,603 (53.81)	13,914 (55.04)	0.0055	0.02
Hypertensive diseases (%)	5923 (19.15)	8951 (25.95)	**<0.0001**	**0.16**	5603 (22.17)	5457 (21.59)	0.1163	0.01
Hyperlipidemia (%)	3241 (10.48)	5087 (14.75)	**<0.0001**	**0.13**	3124 (12.36)	2957 (11.7)	0.0224	0.02
Diabetes mellitus (%)	2908 (9.4)	4275 (12.39)	**<0.0001**	**0.10**	2708 (10.71)	2573 (10.18)	0.0496	0.02
Nicotine dependence (%)	1627 (5.26)	1849 (5.36)	0.5732	0.00	1375 (5.44)	1349 (5.34)	0.6085	0.00
Long term (current) use of systemic steroids (%)	473 (1.53)	708 (2.05)	<0.0001	0.04	436 (1.72)	369 (1.46)	0.0173	0.02
Family history of other specified eye disorder (%)	10 (0.03)	19 (0.06)	0.1677	0.01	10 (0.04)	10 (0.04)	1.0000	0.00

*Std. diff* standard difference, *SD* standard deviation.

**Fig. 1:**
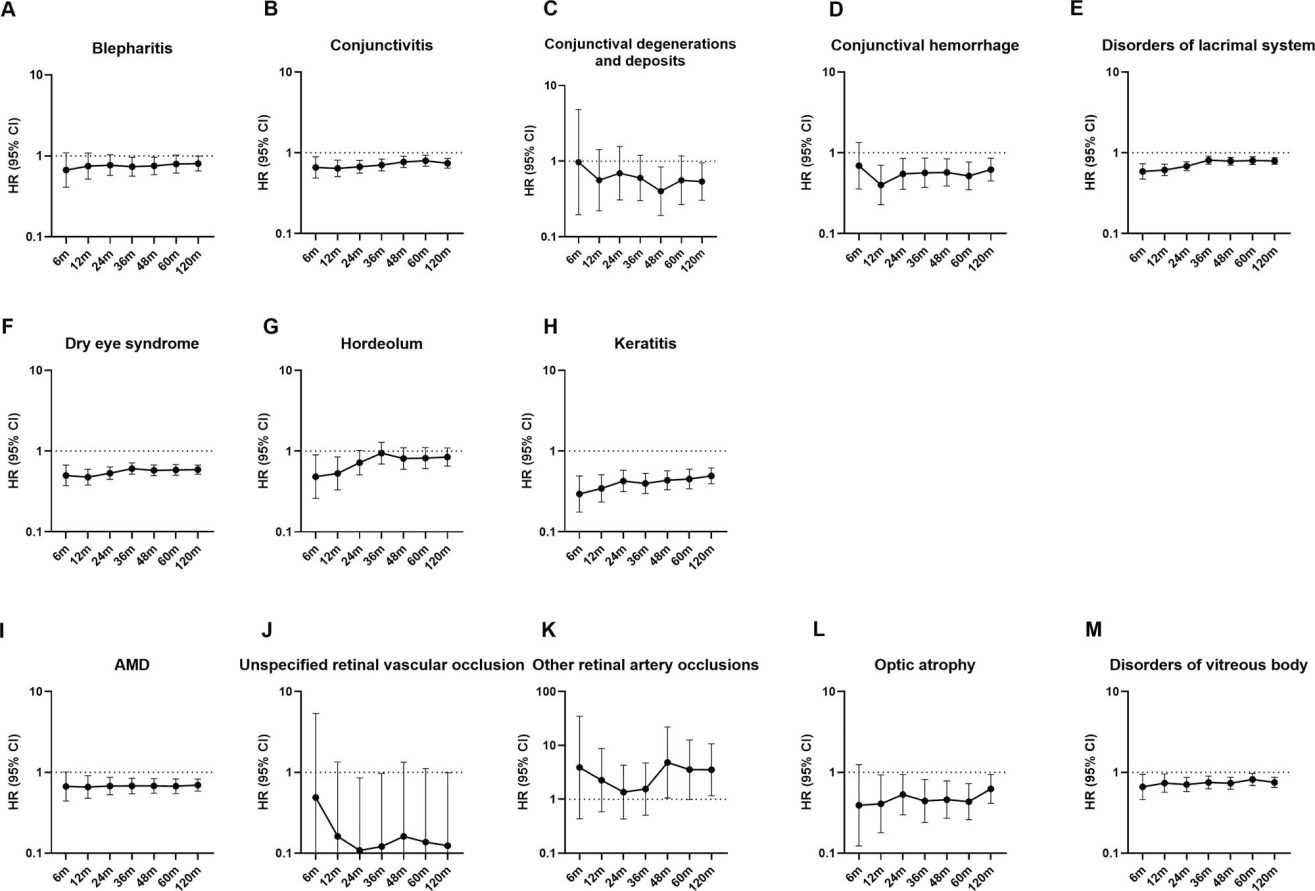
Forest plots of corneal, external eye, retinal, and vitreous disease outcomes in patients with psoriasis treated with biologic versus non-biologic systemic therapies. Forest plots show hazard ratios (HRs) for corneal and external eye diseases (panels **A**–**H**) and retinal and vitreous diseases (panels **I**–**M**) comparing patients with a confirmed diagnosis of psoriasis prescribed biologic agents with those prescribed non-biologic systemic treatments. Points represent HR estimates and horizontal lines indicate 95% confidence intervals. HRs are displayed on a logarithmic scale. AMD age-related macular degeneration.

**Fig. 2:**
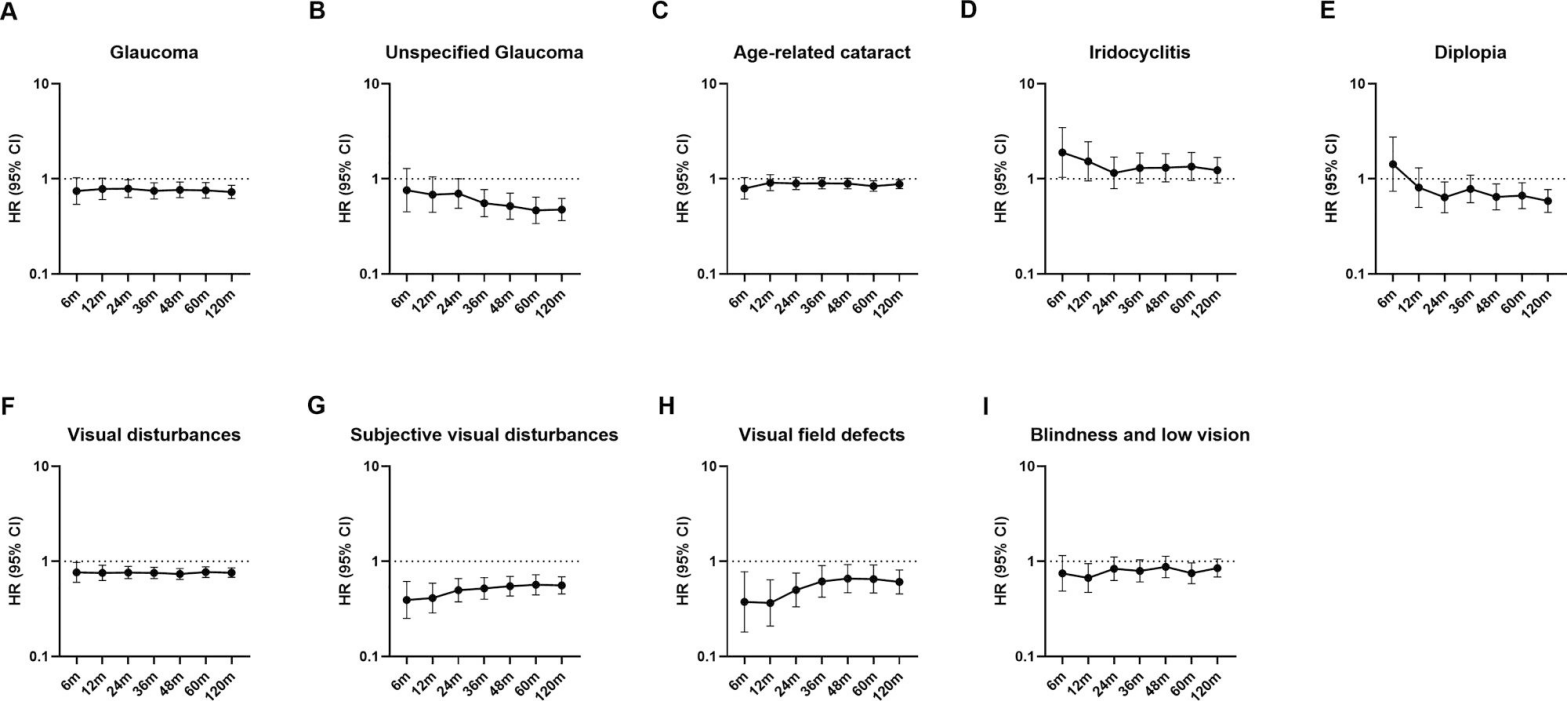
Forest plots of ocular-related outcomes in patients with psoriasis treated with biologic versus non-biologic systemic therapies. Forest plots show hazard ratios (HRs) for glaucoma, cataract, and other ocular-related diagnoses (panels **A**–**I**) comparing patients with a confirmed diagnosis of psoriasis prescribed biologic agents with those prescribed non-biologic systemic treatments. Points represent HR estimates and horizontal lines indicate 95% confidence intervals. HRs are displayed on a logarithmic scale. AMD age-related macular degeneration.

Across all intervals, biologic therapy was consistently associated with lower HRs for corneal and external eye diseases, including keratitis, blepharitis, and conjunctivitis (average HR 0.60), with effects evident from 6 months to 120 months. Lower HRs were also observed for glaucoma and cataract (mean HR ~ 0.88), though results varied by condition. Associations with retinal and vitreous diseases (e.g., AMD, retinal vascular occlusions) were less consistent, with HRs approaching or exceeding 1.0 in longer follow-up. The negative control (inguinal hernia) showed no differences between groups (mean HR 1.07). Full results are provided in Supplementary Table S9, with selected outcomes shown in Figs. 1–2.

### Sensitivity analyses

Analyses were repeated within the United States (US), Europe and Middle East (EMEA), Asia-Pacific (APAC), and Latin America (LATAM) collaborative networks. Only the US and EMEA networks contained sufficient data. We selected nine clinically relevant outcomes for 5-year follow-up: blepharitis, conjunctivitis, keratitis, dry eye syndrome, iridocyclitis, glaucoma, age-related cataract, AMD, and retinal vascular occlusions.

Supplementary Table S10 presents the characteristics of patients with psoriasis treated with biologic agents compared with those treated with systemic non-biologic therapies in the US and EMEA networks. The APAC and LATAM networks did not contain sufficient data to support these analyses.

Across the US network, biologic therapy was associated with significantly lower HRs for most outcomes, including blepharitis (HR 0.75; 95% CI [0.66, 0.85]; *p* < 0.0001), conjunctivitis (HR 0.90; 95% CI [0.83, 0.97]; *p* = 0.0092), keratitis (HR 0.56; 95% CI [0.49, 0.64]; *p* < 0.0001), dry eye syndrome (HR 0.68; 95% CI [0.63, 0.73]; *p* < 0.0001), glaucoma (HR 0.84; 95% CI [0.76, 0.92]; *p* = 0.0004), age-related cataract (HR 0.85; 95% CI [0.80, 0.91]; *p* < 0.0001), and AMD (HR 0.77; 95% CI [0.68, 0.86]; *p* < 0.0001). Iridocyclitis showed a higher HR (HR 1.21; 95% CI [1.00, 1.47]; *p* = 0.0475), and no significant differences were found for retinal vascular occlusions (HR 1.10; 95% CI [0.82, 1.46]; *p* = 0.5355). In the EMEA network, similar but fewer significant associations were observed, including conjunctivitis (HR 0.55, 95% CI [0.39, 0.78]; *p* = 0.0007) and dry eye syndrome (HR 0.64; 95% CI [0.45, 0.91]; *p* = 0.0127). Relevant characteristics and outcomes appear in Supplementary Table S10–S11.

### Assessment of different negative control outcomes

To assess residual confounding, additional negative controls were tested over 5 years. No significant differences were identified for appendicitis (HR 1.113; 95% CI [0.875, 1.416]; *p* = 0.3828), cholelithiasis (HR 0.988; 95% CI [0.909, 1.073]; *p* = 0.7697), dental caries (HR 0.885; 95% CI [0.78, 1.004]; *p* = 0.0574) or wrist/hand fracture (HR 0.956; 95% CI [0.847, 1.079]; *p* = 0.4632), supporting robustness of the primary findings.

### Stratifying outcomes with different pathological mechanisms

To capture differences in pathophysiology, keratitis was stratified by ICD-10 subcodes. Over 5 years, biologic therapy was associated with significantly reduced HRs for corneal ulcer (HR 0.58; 95% CI [0.41, 0.81]; *p* = 0.0013), superficial keratitis without conjunctivitis (HR 0.45; 95% CI [0.35, 0.57]; *p* < 0.0001), keratoconjunctivitis (HR 0.46; 95% CI [0.38, 0.54]; *p* < 0.0001), and corneal neovascularisation (HR 0.44; 95% CI [0.23, 0.85]; *p* = 0.0115). Full results are shown in Supplementary Table S12.

Patients were grouped by therapeutic class (TNF, IL-23, IL-17 inhibitors) and compared pairwise. The baseline characteristics of the comparison groups is summarised in Supplementary Table S13. IL-23 inhibitors showed higher HRs for conjunctivitis (HR = 1.26; 95% CI [1.03, 1.53]; *p* = 0.0215) but lower HRs for iridocyclitis (HR = 0.32; 95% CI [0.18, 0.58]; *p* = 0.0001) compared to TNF inhibitors. IL-17 inhibitors showed higher HRs for conjunctivitis versus TNF inhibitors (HR = 1.21; 95% CI [1.0, 1.46]; *p* = 0.049) and higher HRs for iridocyclitis (HR 2.45; 95% CI [1.27, 4.74]; *p* = 0.0058) but lower HRs for glaucoma (HR 0.64; 95% CI [0.48, 0.87]; *p* = 0.0034) and AMD (HR 0.51; 95% CI [0.35, 0.75]; *p* = 0.0004) versus IL-23 inhibitors (Supplementary Table S14).

### Biologic therapy is associated with lower HR for ophthalmic outcomes in arthropathic psoriasis

Among patients with arthropathic psoriasis (L40.5), biologic therapy remained associated with lower risks of keratitis (HR = 0.70; 95% CI [0.57, 0.87]; *p* = 0.0009), age-related cataract (HR = 0.88; 95% CI [0.80, 0.97]; *p* = 0.011), AMD (HR = 0.72; 95% CI [0.61, 0.84]; *p* = 0.0001), glaucoma (HR = 0.86; 95% CI [0.75, 1.00]; *p* = 0.0499), and dry eye syndrome (HR = 0.83; 95% CI [0.74, 0.92]; *p* = 0.0008), with no significant differences for other outcomes (Supplementary Tables S15–S16).

### Expanded variables for propensity score matching

An extended PSM including additional dermatologic, rheumatologic, gastrointestinal, ocular, and healthcare-utilisation variables produced similar findings. Biologic therapy was associated with lower HRs for blepharitis (HR 0.88; 95% CI [0.82, 0.86]; *p* = 0.0464), conjunctivitis (HR 0.89; 95% CI [0.82, 0.96]; *p* = 0.0032), keratitis (HR 0.63; 95% CI [0.55, 0.72]; *p* < 0.0001), age-related cataract (HR 0.89 [0.83, 0.95]; *p* = 0.0007), AMD (HR 0.74 [0.66, 0.83]; *p* < 0.0001), glaucoma (HR 0.83 [0.75, 0.91]; *p* = 0.0001), and dry eye syndrome (HR 0.68 [0.63, 0.73]; *p* < 0.0001) compared with patients receiving systemic therapy (Supplementary Tables S17–S18).

## Discussion

This large-scale, real-world study provides, to the best of our knowledge, the first comprehensive evaluation of the association between biologic therapy for psoriasis and the subsequent development of ocular comorbidities. Our results suggest that biologic treatment was associated with lower HR for several ocular diseases, particularly those affecting the ocular surface. These findings were consistent across multiple follow-up intervals, from as early as 6 months to 120 months, and were supported by the lack of significant differences in the negative control outcome of inguinal hernia, suggesting that the observed associations were not driven by generalised differences in healthcare utilisation.

The most robust negative associations were observed in diseases of the ocular surface, which are well documented in the literature as common in psoriasis. Mechanistic explanations regarding IL-17 and TNF-α in promoting local inflammation are speculative and intended to provide context rather than confirm causation. For example, in dry eye syndrome, Th1 cells produce IFN-γ, promoting apoptosis and squamous metaplasia of the ocular surface epithelium. Th17 cells and their cytokine IL-17 may disrupt the corneal epithelium, driving the recruitment and activation of neutrophils throughout the ocular surface and lacrimal glands [9, 10]. This activity may partially extend to the conjunctiva, which could explain the lower HR observed in our study [11]. A similar process may occur at the lid margin, where elevated IL-17 levels have been demonstrated in demodex blepharitis [12].

The cornea is a major site in this inflammatory pathway, extending beyond its described role in dry eye syndrome. Elevated levels of IL-17 have been observed in bacterial and viral keratitis [13]. Tear film analysis in keratoconus patients showed elevated levels of cytokines, including IL-17 and TNF-α [14], and similar findings were reported in aqueous humour cytokine analysis from patients with bullous keratopathy [15].

Prior studies have shown an increased prevalence of dry eye disease, blepharitis, and meibomian gland dysfunction in psoriatic patients, often correlating with disease severity and systemic inflammation [1–4]. Our findings are consistent with these observations and extend them by showing that patients receiving biologics had lower HR for such outcomes, although causality cannot be inferred from these results.

The observed lower HR for cataract and glaucoma further supports the hypothesis that systemic inflammation contributes to ocular pathology in psoriasis. Th17-mediated inflammation has been implicated in neurodegenerative changes within the visual pathway and in damage to retinal ganglion cells in glaucoma [16], but these mechanistic links remain speculative in the context of our EMR-based analysis.

While less frequently reported, there is evidence that chronic inflammation, metabolic comorbidities, and corticosteroid use may also contribute to the development of these conditions in psoriatic patients [3, 9]. The attenuation of risk among patients treated with biologics may reflect both the anti-inflammatory effects of these agents and a reduced reliance on corticosteroids, although this remains hypothetical.

In contrast, the relationship between biologic use and retinal or vitreous diseases was more variable. Some conditions, such as AMD and retinal vascular occlusion, did not show consistent protective associations. This may reflect differences in pathophysiological mechanisms, as retinal diseases often involve microvascular or degenerative processes that are less responsive to systemic immunomodulation. Nonetheless, inflammation is also a key factor in the development of both dry and wet AMD, which are associated with an abundance of Th1/Th17 cells and increased secretion of pro-inflammatory cytokines [17, 18]. Further research is warranted to clarify the potential role of biologics in this domain.

Our findings are supported by a growing body of literature showing that early biologic intervention in psoriasis can mitigate systemic comorbidities. Biologics, particularly IL-23 and IL-17 inhibitors, have demonstrated efficacy in reducing cardiovascular risk [5, 6] and the incidence of psoriatic arthritis [7, 8]. The present study adds to this evidence by suggesting that certain ocular comorbidities also demonstrate negative associations with biologic use, although causal interpretations cannot be made.

Several strengths enhance the validity of our study. The use of the TriNetX global federated database enabled the inclusion of large and diverse cohorts with real-world treatment patterns. Propensity score matching was employed to minimise confounding by indication and comorbidities, and multiple follow-up periods allowed for the assessment of both short- and long-term outcomes. Moreover, the use of a negative control outcome helped address concerns regarding residual confounding and selection bias.

Nonetheless, some limitations must be acknowledged. First, diagnoses were based on ICD-10 codes derived from electronic health records, which may be prone to misclassification or underreporting. Second, while we adjusted for key confounders, residual confounding related to disease severity, treatment adherence, and socioeconomic factors cannot be excluded. Third, matching was performed separately for each follow-up interval and for each additional analysis, resulting in multiple distinct matched cohorts. Although this approach may introduce selection and survival bias, it reflects the default matching procedure implemented by the TriNetX platform. Another limitation is the use of broad diagnostic categories that encompass multiple sub-diagnoses. For example, keratitis includes distinct entities such as bacterial and herpes keratitis, each with different pathophysiology. This heterogeneity may bias results and affect the validity of conclusions. Finally, residual confounding due to disease severity is possible, as patients prescribed biologics may have had more severe psoriasis than those on non-biologic systemic treatments. Although direct severity measures were unavailable, we adjusted for proxies of disease burden, including steroid use and comorbidities. Future studies incorporating clinical severity scores would be valuable for further validation.

The use of the TriNetX platform limited the analysis to the tools available within the system, which provide only aggregate, summary-level data derived from predefined query structures. Because patient-level records cannot be accessed or exported, analyses must rely on the platform’s validated modules, such as propensity-score matching and survival modelling. These privacy-preserving constraints preclude the use of more advanced data-science approaches, including machine learning, clustering, or temporal modelling, which require direct manipulation of individual-level longitudinal data. Future studies using datasets that allow secure access to patient-level information may enable the application of these techniques to further characterise treatment-response heterogeneity.

In conclusion, our findings indicate the importance of collaboration between dermatologists, rheumatologists, and ophthalmologists in the care of patients with psoriasis. The negative associations between biologic use and multiple ocular comorbidities identify an area that warrants further mechanistic and prospective investigation. These results provide a rationale for future prospective studies and may influence therapeutic decision-making, particularly in patients with recurrent or sight-threatening ocular inflammation.

Supplemental material is available at Eye’s website.

## Summary

### What is already known about this topic?

- Psoriasis is associated with a wide range of systemic comorbidities, including ocular involvement.
- Biologic therapies reduce cardiovascular and musculoskeletal complications in psoriasis.
- The impact of biologic treatment on ocular comorbidities in psoriasis remains underexplored and poorly characterised in real-world settings.

### What does this study add?

- This large-scale, propensity score-matched cohort study demonstrates that biologic therapy is associated with a significantly lower risk of developing ocular surface disease in psoriasis.
- Protective effects were consistent across multiple follow-up intervals, including long-term observation up to 10 years.
- Findings support consideration of ocular health when planning psoriasis treatment and highlight the systemic benefits of biologic therapy.

## Supplementary information

Supplemental Material Legends

Supplementary Table S1

Supplementary Table S2

Supplementary Table S3

Supplementary Table S4

Supplementary Table S5

Supplementary Table S6

Supplementary Table S7

Supplementary Table S8

Supplementary Table S9

Supplementary Table S10

Supplementary Table S11

Supplementary Table S12

Supplementary Table S13

Supplementary Table S14

Supplementary Table S15

Supplementary Table S16

Supplementary Table S17

Supplementary Table S18

## Data Availability

The data that support the findings of this study were obtained from the TriNetX Global Collaborative Network, a federated health research platform comprising anonymised electronic medical records from multiple healthcare organisations worldwide. Access to the data is subject to licensing restrictions and cannot be shared publicly. Researchers interested in accessing the data used in this study may apply for access directly through TriNetX (https://www.trinetx.com/), subject to institutional approval and data use agreements.
