# Supplementary material for: Biologic therapy is associated with reduced ocular disease in psoriasis: a real-world study
Source: Eye (Lond). 2026 Feb 5;40(5):676–81. doi: 10.1038/s41433-026-04274-x (PMC13013609; doi:10.1038/s41433-026-04274-x)
Supplement: Supplementary file 1 — Supplemental Material Legends [file 41433_2026_4274_MOESM1_ESM.docx]

**Supplementary Material**

**Table S1:** List of biological and non-biological systemic agents for psoriasis with respective International Classification of Diseases, 10th revision (ICD-10) codes. These codes were used for the inclusion criteria.

**Table S2:** List of 68 ocular outcomes measures in both groups with respective International Classification of Diseases, 10th revision (ICD-10) codes. Inguinal hernia was chosen as negative control.

**Table S3:** Patient characteristics before and after propensity score matching for patients with confirmed diagnosis of psoriasis who were prescribed with biologic agents vs. patients with a confirmed diagnosis of psoriasis who were prescribed with non-biologic systemic therapy for a follow-up period of 12 months. Std. diff, standard difference; SD, standard deviation.

**Table S4:** Patient characteristics before and after propensity score matching for patients with confirmed diagnosis of psoriasis who were prescribed with biologic agents vs. patients with a confirmed diagnosis of psoriasis who were prescribed with non-biologic systemic therapy for a follow-up period of 24 months. Std. diff, standard difference; SD, standard deviation.

**Table S5:** Patient characteristics before and after propensity score matching for patients with confirmed diagnosis of psoriasis who were prescribed with biologic agents vs. patients with a confirmed diagnosis of psoriasis who were prescribed with non-biologic systemic therapy for a follow-up period of 36 months. Std. diff, standard difference; SD, standard deviation.

**Table S6:** Patient characteristics before and after propensity score matching for patients with confirmed diagnosis of psoriasis who were prescribed with biologic agents vs. patients with a confirmed diagnosis of psoriasis who were prescribed with non-biologic systemic therapy for a follow-up period of 48 months. Std. diff, standard difference; SD, standard deviation.

**Table S7:** Patient characteristics before and after propensity score matching for patients with confirmed diagnosis of psoriasis who were prescribed with biologic agents vs. patients with a confirmed diagnosis of psoriasis who were prescribed with non-biologic systemic therapy for a follow-up period of 60 months. Std. diff, standard difference; SD, standard deviation.

**Table S8:** Patient characteristics before and after propensity score matching for patients with confirmed diagnosis of psoriasis who were prescribed with biologic agents vs. patients with a confirmed diagnosis of psoriasis who were prescribed with non-biologic systemic therapy for a follow-up period of 120 months. Std. diff, standard difference; SD, standard deviation.

**Table S9:** Summary of hazard ratios (HRs) with 95% confidence interval (95% CI), log-rank tests p-values and proportionality tests p-values for ocular outcomes in patients with confirmed diagnosis of psoriasis who were prescribed with biologic agents vs. patients with a confirmed diagnosis of psoriasis who were prescribed with non-biologic systemic therapy. M, months; HR, hazard ratio; CI, confidence interval.

**Supplementary Table S10:** Characteristics of patients with psoriasis treated with biologic agents compared with those treated with systemic non-biologic therapies in the US and EMEA networks.

**Supplementary Table S11:** Summary of ocular outcomes across the US and EMEA networks in patients with confirmed diagnosis of psoriasis who were prescribed with biologic agents vs. patients with a confirmed diagnosis of psoriasis who were prescribed with non-biologic systemic therapy. Over a follow-up period of 5 years across the US network, biologic therapy was associated with a lower HR for most ophthalmic outcomes compared with systemic non-biologic treatments.

**Supplementary Table S12:** Stratified outcomes with different pathological mechanisms. Patients were grouped based on keratitis subcodes to account for potentially distinct pathophysiological mechanisms. Over a follow-up period of 5 years, several conditions demonstrated statistically significant associations with the exposure.

**Supplementary Table S13:** Baseline characteristics of patients stratified according to therapeutic class. Three groups were defined: patients with psoriasis prescribed TNF inhibitors, IL-23 inhibitors, or IL-17 inhibitors.

**Supplementary Table S14:** Ocular outcomes in patients stratified according to therapeutic class.

**Supplementary Table S15:** Patient characteristics before and after propensity score matching for patients with confirmed diagnosis of arthropathic psoriasis who were prescribed with biologic agents vs. patients with a confirmed diagnosis of arthropathic psoriasis who were prescribed with non-biologic systemic therapy for a follow-up period of 60 months. Std. diff, standard difference; SD, standard deviation.

**Supplementary Table S16:** Summary of hazard ratios (HRs) with 95% confidence interval (95% CI), log-rank tests p-values and proportionality tests p-values for ocular outcomes in patients with confirmed diagnosis of arthropathic psoriasis who were prescribed with biologic agents vs. patients with a confirmed diagnosis of arthropathic psoriasis who were prescribed with non-biologic systemic therapy.

**Supplementary Table S17:** Patient characteristics before and after propensity score matching using an extended list of covariates for patients with confirmed diagnosis of psoriasis who were prescribed with biologic agents vs. patients with a confirmed diagnosis of psoriasis who were prescribed with non-biologic systemic therapy for a follow-up period of 60 months. Std. diff, standard difference; SD, standard deviation.

**Supplementary Table S18:** Summary of hazard ratios (HRs) with 95% confidence interval (95% CI), log-rank tests p-values and proportionality tests p-values for ocular outcomes in patients with confirmed diagnosis of psoriasis who were prescribed with biologic agents vs. patients with a confirmed diagnosis of psoriasis who were prescribed with non-biologic systemic therapy, following an extended version of potential covariates.
