# Supplementary material for: Biologic therapy is associated with reduced ocular disease in psoriasis: a real-world study
Source: Eye (Lond). 2026 Feb 5;40(5):676–81. doi: 10.1038/s41433-026-04274-x (PMC13013609; doi:10.1038/s41433-026-04274-x)
Supplement: Supplementary file 2 — Supplementary Table S1 [file 41433_2026_4274_MOESM2_ESM.pdf]

		**ICD-10\RxNorm code**
Psoriasis		L40
TNF inhibitor	Adalimumab	327361
	Etanercept	214555
	Infliximab	191831
	Cetrolizumab	709271
IL-12/23 inhibitor	Ustekinumab	847083
IL-17A inhibitor	Secukinumab	1599788
	Ixekizumab	1745099
IL-17A/F inhibitor	Bimekizumab	2668041
IL-23 inhibitor	Guselkumab	1928588
	Tildrakizumab	2053436
	Risankizumab	2166040
Systemic agents	Methotrexate	6851
	Cyclosporine	3008
	Acitretin	16818
	Apremilast	1492727
	Dimethyl Fumarate	1373478
