# Supplementary material for: Biologic therapy is associated with reduced ocular disease in psoriasis: a real-world study
Source: Eye (Lond). 2026 Feb 5;40(5):676–81. doi: 10.1038/s41433-026-04274-x (PMC13013609; doi:10.1038/s41433-026-04274-x)
Supplement: Supplementary file 3 — Supplementary Table S2 [file 41433_2026_4274_MOESM3_ESM.pdf]

| **Diagnosis** | **ICD-10 code** |
|---|---|
| Hordeolum (externum) (internum) of eyelid | H00.0 |
| Chalazion | H00.1 |
| Blepharitis | H01.0 |
| Disorders of lacrimal system | H00-H05 |
| Acute inflammation of orbit | H05.0 |
| Chronic inflammatory disorders of orbit | H05.1 |
| Conjunctivitis | H10 |
| Pterygium of eye | H11.0 |
| Conjunctival degenerations and deposits | H11.1 |
| Conjunctival scars | H11.2 |
| Conjunctival hemorrhage | H11.4 |
| Other conjunctival vascular disorders and cysts | H11.4 |
| Scleritis | H15.0 |
| Episcleritis | H15.1 |
| Keratitis | H16 |
| Corneal pigmentations and deposits | H18.1 |
| Bullous keratopathy | H18.4 |
| Corneal degeneration | H18.4 |
| Keratoconus | H18.6 |
| Iridocyclitis | H20 |
| Age-related cataract | H25 |
| Chorioretinal inflammation | H30 |
| Other disorders of choroid | H31 |
| Retinal detachment with retinal break | H33.0 |
| Retinoschisis and retinal cysts | H33.1 |
| Serous retinal detachment | H33.2 |
| Retinal breaks without detachment | H33.3 |
| Traction detachment of retina | H33.4 |
| Retinal vascular occlusions | H34 |
| Transient retinal artery occlusion | H34.0 |
| Central retinal artery occlusion | H34.1 |
| Other retinal artery occlusions | H34.2 |
| Other retinal vascular occlusions | H34.8 |
| Unspecified retinal vascular occlusion | H34.9 |
| Type 1 diabetes mellitus with ophthalmic complications | E10.3 |
| Type 2 diabetes mellitus with ophthalmic complications | E11.3 |
| Other specified diabetes mellitus with ophthalmic complications | E13.3 |
| Background retinopathy and retinal vascular changes | H35.0 |
| Other retinal detachments | H33.8 |
| AMD | H35.8 |
| nvAMD | H35.32 |

| | |
|---|---|
| non-nvAMD | H35.31 |
| Glaucoma | H40-H42 |
| Glaucoma suspect | H40.0 |
| Open-angle glaucoma | H40.1 |
| Primary angle-closure glaucoma | H40.2 |
| Glaucoma secondary to eye trauma | H40.3 |
| Glaucoma secondary to eye inflammation | H40.4 |
| Glaucoma secondary to other eye disorders | H40.5 |
| Glaucoma secondary to drugs | H40.6 |
| Other glaucoma | H40.8 |
| Unspecified glaucoma | H40.9 |
| Disorders of vitreous body | H43 |
| Disorders of globe | H44 |
| Optic neuritis | H46 |
| Ischemic optic neuropathy | H47.01 |
| Optic atrophy | H47.2 |
| Paralytic strabismus | H49 |
| Third oculomotor nerve palsy | H49.0 |
| Dry eye syndrome | H04.12 |
| Diplopia | H53.2 |
| Visual disturbances | H53 |
| Blindness and low vision | H54 |
| Intraoperative and postprocedural complications and disorders of eye and adnexa, not elsewhere classified | H59 |
| Visual field defects | H53.4 |
| Subjective visual disturbances | H53.1 |
| Conjunctival hyperemia | H11.43 |
| Color vision deficiencies | H53.5 |
| Inguinal Hernia | K40 |
