# Supplementary material for: Biologic therapy is associated with reduced ocular disease in psoriasis: a real-world study
Source: Eye (Lond). 2026 Feb 5;40(5):676–81. doi: 10.1038/s41433-026-04274-x (PMC13013609; doi:10.1038/s41433-026-04274-x)
Supplement: Supplementary file 4 — Supplementary Table S3 [file 41433_2026_4274_MOESM4_ESM.pdf]

Characteristic Name	Before PSM				After PSM			
	**Biological (n=30,991)**	**Systemic (n=35,832)**	***P***	**Std diff.**	**Biological (n=25,278)**	**Systemic (n=25,278)**	***P***	**Std diff.**
Age at Index (mean ± SD)	47.71±17.04	54.54±17.42	**<0.0001**	**0.40**	51.04±16.23	50.45±17.0	<0.0001	0.04
White (%)	22612 (73.11)	20484 (59.38)	**<0.0001**	**0.29**	17193 (68.02)	16704 (66.08)	<0.0001	0.04
Female (%)	16293 (52.68)	19088 (55.33)	<0.0001	0.05	13603 (53.81)	13914 (55.04)	0.0055	0.02
Hypertensive diseases (%)	5923 (19.15)	8951 (25.95)	**<0.0001**	**0.16**	5603 (22.17)	5457 (21.59)	0.1163	0.01
Hyperlipidemia (%)	3241 (10.48)	5087 (14.75)	**<0.0001**	**0.13**	3124 (12.36)	2957 (11.7)	0.0224	0.02
Diabetes mellitus (%)	2908 (9.4)	4275 (12.39)	<0.0001	0.10	2708 (10.71)	2573 (10.18)	0.0496	0.02
Nicotine dependence (%)	1627 (5.26)	1849 (5.36)	0.5732	0.00	1375 (5.44)	1349 (5.34)	0.6085	0.00
Long term (current) use of systemic steroids (%)	473 (1.53)	708 (2.05)	<0.0001	0.04	436 (1.72)	369 (1.46)	0.0173	0.02
Family history of other specified eye disorder (%)	10 (0.03)	19 (0.06)	0.1677	0.01	10 (0.04)	10 (0.04)	1.0000	0.00
