# Supplementary material for: Biologic therapy is associated with reduced ocular disease in psoriasis: a real-world study
Source: Eye (Lond). 2026 Feb 5;40(5):676–81. doi: 10.1038/s41433-026-04274-x (PMC13013609; doi:10.1038/s41433-026-04274-x)
Supplement: Supplementary file 5 — Supplementary Table S4 [file 41433_2026_4274_MOESM5_ESM.pdf]

Characteristic Name	Before PSM				After PSM			
	**Biological (n=30,955)**	**Systemic (n=35,786)**	***P***	**Std diff.**	**Biological (n=25,239)**	**Systemic (n=25,239)**	***P***	**Std diff.**
Age at Index (mean ± SD)	47.7±17.04	54.53±17.42	**<0.0001**	**0.40**	51.02±16.22	50.43±17.0	<0.0001	0.04
White (%)	22582 (73.1)	20446 (59.35)	**<0.0001**	**0.29**	17163 (68.0)	16692 (66.14)	<0.0001	0.04
Female (%)	16275 (52.68)	19054 (55.31)	<0.0001	0.05	13594 (53.86)	13961 (55.32)	0.00104	0.03
Hypertensive diseases (%)	5915 (19.15)	8932 (25.93)	**<0.0001**	**0.16**	5593 (22.16)	5437 (21.54)	0.09291	0.01
Hyperlipidemia (%)	3240 (10.49)	5083 (14.75)	**<0.0001**	**0.13**	3132 (12.41)	3025 (11.98)	0.14559	0.01
Diabetes mellitus (%)	2905 (9.4)	4261 (12.37)	<0.0001	0.10	2695 (10.68)	2577 (10.21)	0.08592	0.02
Nicotine dependence (%)	1626 (5.26)	1849 (5.37)	0.55718	0.00	1400 (5.55)	1358 (5.38)	0.41077	0.01
Long term (current) use of systemic steroids (%)	473 (1.53)	707 (2.05)	<0.0001	0.04	437 (1.73)	360 (1.43)	0.00597	0.02
Family history of other specified eye disorder (%)	10 (0.03)	19 (0.06)	0.16754	0.01	10 (0.04)	10 (0.04)	1	0.00
