# Supplementary material for: Biologic therapy is associated with reduced ocular disease in psoriasis: a real-world study
Source: Eye (Lond). 2026 Feb 5;40(5):676–81. doi: 10.1038/s41433-026-04274-x (PMC13013609; doi:10.1038/s41433-026-04274-x)
Supplement: Supplementary file 6 — Supplementary Table S5 [file 41433_2026_4274_MOESM6_ESM.pdf]

Characteristic Name	Before PSM				After PSM			
	**Biological (n=29,768)**	**Systemic (n=33,767)**	***P***	**Std diff.**	**Biological (n=23,900)**	**Systemic (n=23,900)**	***P***	**Std diff.**
Age at Index (mean ± SD)	47.65±17.07	54.43±17.49	**<0.0001**	**0.39**	51.08±16.35	50.44±17.04	<0.0001	0.04
White (%)	21446 (72.2)	18499 (57.04)	**<0.0001**	**0.32**	15862 (66.37)	15400 (64.44)	<0.0001	0.04
Female (%)	15572 (52.42)	17767 (54.78)	<0.0001	0.05	12809 (53.59)	13139 (54.97)	0.0024	0.03
Hypertensive diseases (%)	5491 (18.49)	7976 (24.59)	**<0.0001**	**0.15**	5131 (21.47)	4920 (20.59)	0.0179	0.02
Hyperlipidemia (%)	2949 (9.93)	4395 (13.55)	**<0.0001**	**0.11**	2813 (11.77)	2622 (10.97)	0.0059	0.03
Diabetes mellitus (%)	2664 (8.97)	3793 (11.7)	<0.0001	0.09	2452 (10.26)	2272 (9.51)	0.0058	0.03
Nicotine dependence (%)	1424 (4.79)	1504 (4.64)	0.3571	0.01	1169 (4.89)	1124 (4.7)	0.3355	0.01
Long term (current) use of systemic steroids (%)	402 (1.35)	560 (1.73)	0.0002	0.03	364 (1.52)	309 (1.29)	0.0327	0.02
Family history of other specified eye disorder (%)	10 (0.03)	15 (0.05)	0.4346	0.01	10 (0.04)	10 (0.04)	1.0000	0.00
