# Supplementary material for: Biologic therapy is associated with reduced ocular disease in psoriasis: a real-world study
Source: Eye (Lond). 2026 Feb 5;40(5):676–81. doi: 10.1038/s41433-026-04274-x (PMC13013609; doi:10.1038/s41433-026-04274-x)
Supplement: Supplementary file 8 — Supplementary Table S7 [file 41433_2026_4274_MOESM8_ESM.pdf]

| Characteristic Name | Before PSM | | | | After PSM | | | |
|---|---|---|---|---|---|---|---|---|
| | **Biological (n=25,150)** | **Systemic (n=31,143)** | ***P*** | **Std diff.** | **Biological (n=20,905)** | **Systemic (n=20,905)** | ***P*** | **Std diff.** |
| Age at Index (mean ± SD) | 47.98±16.97 | 54.39±17.4 | **<0.0001** | **0.37** | 50.98±16.28 | 50.48±17.0 | 0.0021 | 0.03 |
| White (%) | 17984 (71.66) | 16872 (56.51) | **<0.0001** | **0.32** | 13907 (66.53) | 13623 (65.17) | 0.0034 | 0.03 |
| Female (%) | 13292 (52.96) | 16304 (54.61) | 0.0001 | 0.03 | 11240 (53.77) | 11590 (55.44) | 0.0006 | 0.03 |
| Hypertensive diseases (%) | 4674 (18.62) | 7393 (24.76) | **<0.0001** | **0.15** | 4461 (21.34) | 4401 (21.05) | 0.4728 | 0.01 |
| Hyperlipidemia (%) | 2603 (10.37) | 4203 (14.08) | **<0.0001** | **0.11** | 2531 (12.11) | 2432 (11.63) | 0.1344 | 0.01 |
| Diabetes mellitus (%) | 2318 (9.24) | 3595 (12.04) | <0.0001 | 0.09 | 2179 (10.42) | 2088 (9.99) | 0.1415 | 0.01 |
| Nicotine dependence (%) | 1295 (5.16) | 1543 (5.17) | 0.9657 | 0.00 | 1123 (5.37) | 1098 (5.25) | 0.5856 | 0.01 |
| Long term (current) use of systemic steroids (%) | 442 (1.76) | 664 (2.22) | 0.0001 | 0.03 | 409 (1.96) | 360 (1.72) | 0.0745 | 0.02 |
| Family history of other specified eye disorder (%) | 10 (0.04) | 16 (0.05) | 0.4605 | 0.01 | 10 (0.05) | 11 (0.05) | 0.8272 | 0.00 |
