# Supplementary material for: Biologic therapy is associated with reduced ocular disease in psoriasis: a real-world study
Source: Eye (Lond). 2026 Feb 5;40(5):676–81. doi: 10.1038/s41433-026-04274-x (PMC13013609; doi:10.1038/s41433-026-04274-x)
Supplement: Supplementary file 9 — Supplementary Table S8 [file 41433_2026_4274_MOESM9_ESM.pdf]

Characteristic Name	Before PSM				After PSM			
	**Biological (n=27,344)**	**Systemic (n=32,836)**	***P***	**Std diff.**	**Biological (n=22,617)**	**Systemic (n=22,617)**	***P***	**Std diff.**
Age at Index (mean ± SD)	47.86±16.98	54.48±17.39	**<0.0001**	**0.39**	50.99±16.2	50.33±16.94	<0.0001	0.04
White (%)	19592 (71.79)	18137 (57.49)	**<0.0001**	**0.30**	15105 (66.79)	14606 (64.58)	<0.0001	0.05
Female (%)	14426 (52.86)	17358 (55.02)	<0.0001	0.04	12240 (54.12)	12420 (54.91)	0.0892	0.02
Hypertensive diseases (%)	5113 (18.74)	7910 (25.07)	**<0.0001**	**0.15**	4877 (21.56)	4700 (20.78)	0.0416	0.02
Hyperlipidemia (%)	2777 (10.18)	4448 (14.1)	**<0.0001**	**0.12**	2691 (11.9)	2553 (11.29)	0.0427	0.02
Diabetes mellitus (%)	2532 (9.28)	3823 (12.12)	<0.0001	0.09	2365 (10.46)	2315 (10.24)	0.4402	0.01
Nicotine dependence (%)	1393 (5.1)	1637 (5.19)	0.6437	0.00	1203 (5.32)	1159 (5.12)	0.3524	0.01
Long term (current) use of systemic steroids (%)	442 (1.62)	664 (2.1)	<0.0001	0.04	403 (1.78)	354 (1.57)	0.0725	0.02
Family history of other specified eye disorder (%)	10 (0.04)	16 (0.05)	0.4179	0.01	10 (0.04)	10 (0.04)	1.0000	0.00
