# Supplementary material for: Biologic therapy is associated with reduced ocular disease in psoriasis: a real-world study
Source: Eye (Lond). 2026 Feb 5;40(5):676–81. doi: 10.1038/s41433-026-04274-x (PMC13013609; doi:10.1038/s41433-026-04274-x)
Supplement: Supplementary file 10 — Supplementary Table S9 [file 41433_2026_4274_MOESM10_ESM.pdf]

| Follow-up | Outcome | Patients in cohort | | Patients with outcome | | HR [95% CI] | Log-rank test p-value | Proportionality test p-value |
|---|---|---|---|---|---|---|---|---|
| | | Biologic | Systemic | Biologic | Systemic | | | |
| 6m | Hordeolum (externum) (internum) of eyelid | 25102 | 24987 | 15 | 30 | 0.48 [0.26, 0.9] | 0.0191 | 0.2259 |
| 12m | Hordeolum (externum) (internum) of eyelid | 25102 | 24987 | 27 | 49 | 0.53 [0.33, 0.85] | 0.0072 | 0.7395 |
| 24m | Hordeolum (externum) (internum) of eyelid | 25069 | 24937 | 55 | 74 | 0.72 [0.51, 1.02] | 0.0633 | 0.2727 |
| 36m | Hordeolum (externum) (internum) of eyelid | 23740 | 23623 | 80 | 82 | 0.94 [0.69, 1.29] | 0.7169 | 0.0301 |
| 48m | Hordeolum (externum) (internum) of eyelid | 22467 | 22363 | 76 | 90 | 0.81 [0.6, 1.1] | 0.179 | 0.0172 |
| 60m | Hordeolum (externum) (internum) of eyelid | 20767 | 20667 | 79 | 93 | 0.82 [0.61, 1.11] | 0.1953 | 0.4741 |
| 120m | Hordeolum (externum) (internum) of eyelid | 22467 | 22363 | 108 | 127 | 0.85 [0.66, 1.09] | 0.2039 | 0.3405 |
| 6m | Chalazion | 25184 | 25169 | 10 | 10 | 0.58 [0.21, 1.6] | 0.2887 | 0.2292 |
| 12m | Chalazion | 25184 | 25169 | 13 | 15 | 0.84 [0.4, 1.76] | 0.6395 | 0.9114 |
| 24m | Chalazion | 25147 | 25123 | 23 | 29 | 0.77 [0.45, 1.33] | 0.3529 | 0.0622 |
| 36m | Chalazion | 23812 | 23810 | 31 | 26 | 1.17 [0.69, 1.96] | 0.565 | 0.5621 |
| 48m | Chalazion | 22539 | 22513 | 41 | 33 | 1.2 [0.76, 1.9] | 0.4302 | 0.0737 |
| 60m | Chalazion | 20835 | 20808 | 42 | 28 | 1.46 [0.9, 2.35] | 0.1203 | 0.4471 |
| 120m | Chalazion | 22539 | 22513 | 56 | 50 | 1.13 [0.77, 1.66] | 0.525 | 0.3878 |

| | | | | | | | | |
|---|---|---|---|---|---|---|---|---|
| 6m | Blepharitis | 25008 | 24794 | 27 | 39 | 0.67 [0.41, 1.09] | 0.1052 | 0.0561 |
| 12m | Blepharitis | 25008 | 24794 | 50 | 64 | 0.75 [0.52, 1.08] | 0.1251 | 0.857 |
| 24m | Blepharitis | 24967 | 24738 | 80 | 100 | 0.77 [0.57, 1.03] | 0.0806 | 0.43 |
| 36m | Blepharitis | 23647 | 23448 | 95 | 124 | 0.74 [0.56, 0.96] | 0.0247 | 0.9868 |
| 48m | Blepharitis | 22384 | 22182 | 109 | 138 | 0.75 [0.59, 0.97] | 0.0271 | 0.6853 |
| 60m | Blepharitis | 20702 | 20518 | 108 | 130 | 0.8 [0.62, 1.03] | 0.0783 | 0.6037 |
| 120m | Blepharitis | 22384 | 22182 | 160 | 196 | 0.81 [0.65, 0.99] | 0.0431 | 0.839 |
| 6m | Disorders of lacrimal system | 23932 | 23055 | 132 | 209 | 0.59 [0.47, 0.73] | <0.0001 | 0.6711 |
| 12m | Disorders of lacrimal system | 23932 | 23055 | 238 | 358 | 0.61 [0.52, 0.72] | <0.0001 | 0.7072 |
| 24m | Disorders of lacrimal system | 23916 | 22963 | 414 | 558 | 0.68 [0.6, 0.78] | <0.0001 | 0.0164 |
| 36m | Disorders of lacrimal system | 22659 | 21847 | 545 | 621 | 0.81 [0.72, 0.91] | 0.0003 | 0 |
| 48m | Disorders of lacrimal system | 21469 | 20596 | 587 | 676 | 0.79 [0.71, 0.88] | <0.0001 | 0.0007 |
| 60m | Disorders of lacrimal system | 19894 | 19147 | 574 | 654 | 0.8 [0.72, 0.9] | 0.0001 | 0.1612 |
| 120m | Disorders of lacrimal system | 21469 | 20596 | 795 | 932 | 0.8 [0.72, 0.87] | <0.0001 | 0.0746 |
| 6m | Acute inflammation of orbit | 25265 | 25246 | 0 | 10 | nan [nan, nan] | 0.1515 | nan |
| 12m | Acute inflammation of orbit | 25265 | 25246 | 0 | 10 | nan [nan, nan] | 0.0229 | nan |

| | | | | | | | | |
|---|---|---|---|---|---|---|---|---|
| 24m | Acute inflammation of orbit | 25227 | 25209 | 0 | 10 | nan [nan, nan] | 0.0073 | nan |
| 36m | Acute inflammation of orbit | 23889 | 23876 | 10 | 10 | 0.19 [0.02, 1.67] | 0.0956 | 0.0665 |
| 48m | Acute inflammation of orbit | 22603 | 22588 | 10 | 10 | 0.28 [0.06, 1.33] | 0.0858 | 0.3574 |
| 60m | Acute inflammation of orbit | 20892 | 20880 | 10 | 10 | 0.1 [0.01, 0.76] | 0.0056 | 0.9786 |
| 120m | Acute inflammation of orbit | 22603 | 22588 | 10 | 10 | 0.63 [0.21, 1.92] | 0.4095 | 0.0137 |
| 6m | Chronic inflammatory disorders of orbit | 25267 | 25264 | 0 | 0 | nan [nan, nan] | 1 | nan |
| 12m | Chronic inflammatory disorders of orbit | 25267 | 25264 | 0 | 0 | nan [nan, nan] | 1 | nan |
| 24m | Chronic inflammatory disorders of orbit | 25229 | 25226 | 10 | 10 | 0.49 [0.04, 5.41] | 0.5523 | 0.0997 |
| 36m | Chronic inflammatory disorders of orbit | 23892 | 23888 | 10 | 10 | 0.65 [0.11, 3.89] | 0.6345 | 0.5867 |
| 48m | Chronic inflammatory disorders of orbit | 22609 | 22607 | 10 | 10 | 0.32 [0.03, 3.09] | 0.3002 | 0.6485 |
| 60m | Chronic inflammatory disorders of orbit | 20897 | 20896 | 0 | 10 | nan [nan, nan] | 0.0776 | nan |
| 120m | Chronic inflammatory disorders of orbit | 22609 | 22607 | 10 | 10 | 0.5 [0.09, 2.75] | 0.4191 | 0.6915 |
| 6m | Conjunctivitis | 24466 | 24037 | 71 | 103 | 0.66 [0.49, 0.89] | 0.0065 | 0.0738 |
| 12m | Conjunctivitis | 24466 | 24037 | 119 | 176 | 0.64 [0.51, 0.81] | 0.0002 | 0.1796 |
| 24m | Conjunctivitis | 24417 | 23926 | 201 | 284 | 0.67 [0.56, 0.81] | <0.0001 | 0.416 |
| 36m | Conjunctivitis | 23164 | 22724 | 244 | 327 | 0.71 [0.6, 0.84] | <0.0001 | 0.0905 |

| | | | | | | | | |
|---|---|---|---|---|---|---|---|---|
| 48m | Conjunctivitis | 21935 | 21510 | 276 | 336 | 0.77 [0.66, 0.91] | 0.0014 | 0.1326 |
| 60m | Conjunctivitis | 20252 | 19863 | 287 | 340 | 0.8 [0.68, 0.93] | 0.0048 | 0.7276 |
| 120m | Conjunctivitis | 21935 | 21510 | 366 | 477 | 0.74 [0.65, 0.85] | <0.0001 | 0.861 |
| 6m | Pterygium of eye | 25256 | 25233 | 10 | 10 | 0.24 [0.03, 2.17] | 0.1697 | 0.1061 |
| 12m | Pterygium of eye | 25256 | 25233 | 10 | 10 | 0.36 [0.1, 1.37] | 0.1176 | 0.2154 |
| 24m | Pterygium of eye | 25217 | 25188 | 10 | 13 | 0.3 [0.1, 0.92] | 0.025 | 0.4587 |
| 36m | Pterygium of eye | 23878 | 23859 | 10 | 16 | 0.37 [0.14, 0.93] | 0.0281 | 0.8694 |
| 48m | Pterygium of eye | 22598 | 22575 | 10 | 15 | 0.58 [0.25, 1.33] | 0.1924 | 0.6987 |
| 60m | Pterygium of eye | 20891 | 20872 | 10 | 14 | 0.62 [0.27, 1.44] | 0.2647 | 0.3297 |
| 120m | Pterygium of eye | 22598 | 22575 | 14 | 17 | 0.82 [0.4, 1.66] | 0.5784 | 0.0779 |
| 6m | Conjunctival degenerations and deposits | 25242 | 25211 | 10 | 10 | 0.97 [0.2, 4.81] | 0.9705 | 0.7612 |
| 12m | Conjunctival degenerations and deposits | 25242 | 25211 | 10 | 12 | 0.56 [0.22, 1.43] | 0.2195 | 0.1996 |
| 24m | Conjunctival degenerations and deposits | 25206 | 25183 | 10 | 14 | 0.69 [0.31, 1.56] | 0.3739 | 0.401 |
| 36m | Conjunctival degenerations and deposits | 23870 | 23847 | 13 | 21 | 0.6 [0.3, 1.2] | 0.1457 | 0.9983 |
| 48m | Conjunctival degenerations and deposits | 22583 | 22562 | 10 | 24 | 0.4 [0.19, 0.84] | 0.0121 | 0.7786 |
| 60m | Conjunctival degenerations and deposits | 20876 | 20856 | 11 | 19 | 0.56 [0.27, 1.18] | 0.1197 | 0.4378 |

| | | | | | | | | |
|---|---|---|---|---|---|---|---|---|
| 120m | Conjunctival degenerations and deposits | 22583 | 22562 | 18 | 34 | 0.54 [0.3, 0.95] | 0.0314 | 0.7567 |
| 6m | Conjunctival scars | 25273 | 25266 | 0 | 10 | nan [nan, nan] | 0.0788 | nan |
| 12m | Conjunctival scars | 25273 | 25266 | 0 | 10 | nan [nan, nan] | 0.0422 | nan |
| 24m | Conjunctival scars | 25233 | 25229 | 10 | 10 | 0.16 [0.02, 1.35] | 0.0548 | 0.8765 |
| 36m | Conjunctival scars | 23895 | 23891 | 10 | 10 | 0.16 [0.02, 1.34] | 0.0537 | 0.3013 |
| 48m | Conjunctival scars | 22612 | 22607 | 10 | 10 | 0.24 [0.05, 1.14] | 0.0521 | 0.4815 |
| 60m | Conjunctival scars | 20901 | 20898 | 10 | 10 | 0.28 [0.06, 1.34] | 0.0878 | 0.4396 |
| 120m | Conjunctival scars | 22612 | 22607 | 10 | 10 | 0.3 [0.08, 1.09] | 0.0529 | 0.3014 |
| 6m | Conjunctival hemorrhage | 25188 | 25128 | 15 | 21 | 0.69 [0.36, 1.34] | 0.2756 | 0.2685 |
| 12m | Conjunctival hemorrhage | 25188 | 25128 | 17 | 41 | 0.4 [0.23, 0.7] | 0.001 | 0.0229 |
| 24m | Conjunctival hemorrhage | 25151 | 25088 | 31 | 55 | 0.55 [0.35, 0.85] | 0.0066 | 0.7957 |
| 36m | Conjunctival hemorrhage | 23828 | 23763 | 35 | 60 | 0.57 [0.37, 0.86] | 0.0066 | 0.6449 |
| 48m | Conjunctival hemorrhage | 22540 | 22479 | 41 | 69 | 0.57 [0.39, 0.84] | 0.0042 | 0.7109 |
| 60m | Conjunctival hemorrhage | 20836 | 20784 | 38 | 71 | 0.52 [0.35, 0.77] | 0.0009 | 0.9332 |
| 120m | Conjunctival hemorrhage | 22540 | 22479 | 60 | 97 | 0.62 [0.45, 0.86] | 0.0034 | 0.7548 |
| 6m | Other conjunctival vascular disorders and cysts | 25244 | 25224 | 10 | 10 | 0.49 [0.12, 1.96] | 0.3015 | 0.3196 |

| | | | | | | | | |
|---|---|---|---|---|---|---|---|---|
| 12m | Other conjunctival vascular disorders and cysts | 25244 | 25224 | 10 | 10 | 0.39 [0.12, 1.24] | 0.0976 | 0.6684 |
| 24m | Other conjunctival vascular disorders and cysts | 25201 | 25182 | 10 | 14 | 0.35 [0.13, 0.96] | 0.0337 | 0.7831 |
| 36m | Other conjunctival vascular disorders and cysts | 23870 | 23856 | 10 | 12 | 0.73 [0.31, 1.73] | 0.4721 | 0.0348 |
| 48m | Other conjunctival vascular disorders and cysts | 22587 | 22568 | 10 | 15 | 0.65 [0.29, 1.44] | 0.2801 | 0.6405 |
| 60m | Other conjunctival vascular disorders and cysts | 20877 | 20859 | 10 | 16 | 0.61 [0.28, 1.35] | 0.2169 | 0.5677 |
| 120m | Other conjunctival vascular disorders and cysts | 22587 | 22568 | 17 | 21 | 0.82 [0.43, 1.56] | 0.5456 | 0.7639 |
| 6m | Scleritis | 25225 | 25224 | 10 | 10 | 0.19 [0.02, 1.67] | 0.0959 | 0.2157 |
| 12m | Scleritis | 25225 | 25224 | 10 | 10 | 0.58 [0.14, 2.44] | 0.454 | 0.2766 |
| 24m | Scleritis | 25184 | 25184 | 10 | 12 | 0.49 [0.18, 1.3] | 0.1421 | 0.7069 |
| 36m | Scleritis | 23851 | 23856 | 10 | 14 | 0.56 [0.23, 1.33] | 0.1808 | 0.7673 |
| 48m | Scleritis | 22567 | 22565 | 10 | 15 | 0.58 [0.26, 1.33] | 0.1956 | 0.8019 |
| 60m | Scleritis | 20865 | 20851 | 11 | 15 | 0.71 [0.33, 1.56] | 0.3948 | 0.6938 |
| 120m | Scleritis | 22567 | 22565 | 18 | 19 | 0.96 [0.51, 1.84] | 0.9105 | 0.0274 |
| 6m | Episcleritis | 25225 | 25223 | 10 | 10 | 0.56 [0.16, 1.9] | 0.343 | 0.8895 |
| 12m | Episcleritis | 25225 | 25223 | 10 | 10 | 0.85 [0.31, 2.34] | 0.7524 | 0.4197 |
| 24m | Episcleritis | 25183 | 25188 | 10 | 10 | 1.08 [0.44, 2.66] | 0.8624 | 0.2501 |

| | | | | | | | | |
|---|---|---|---|---|---|---|---|---|
| 36m | Episcleritis | 23848 | 23851 | 10 | 13 | 0.75 [0.33, 1.71] | 0.4912 | 0.2247 |
| 48m | Episcleritis | 22567 | 22572 | 11 | 15 | 0.71 [0.33, 1.55] | 0.3859 | 0.8005 |
| 60m | Episcleritis | 20865 | 20859 | 12 | 17 | 0.69 [0.33, 1.44] | 0.3143 | 0.4216 |
| 120m | Episcleritis | 22567 | 22572 | 16 | 19 | 0.83 [0.43, 1.62] | 0.5936 | 0.6814 |
| 6m | Keratitis | 25033 | 24732 | 19 | 62 | 0.29 [0.18, 0.49] | <0.0001 | 0.3257 |
| 12m | Keratitis | 25033 | 24732 | 35 | 97 | 0.34 [0.23, 0.51] | <0.0001 | 0.2362 |
| 24m | Keratitis | 24990 | 24661 | 60 | 135 | 0.43 [0.31, 0.58] | <0.0001 | 0.5991 |
| 36m | Keratitis | 23682 | 23404 | 66 | 159 | 0.4 [0.3, 0.53] | <0.0001 | 0.6782 |
| 48m | Keratitis | 22390 | 22083 | 77 | 168 | 0.43 [0.33, 0.57] | <0.0001 | 0.6313 |
| 60m | Keratitis | 20710 | 20479 | 72 | 152 | 0.45 [0.34, 0.6] | <0.0001 | 0.209 |
| 120m | Keratitis | 22390 | 22083 | 113 | 224 | 0.49 [0.39, 0.62] | <0.0001 | 0.4041 |
| 6m | Corneal pigmentations and deposits | 25265 | 25274 | 0 | 10 | nan [nan, nan] | 0.3148 | nan |
| 12m | Corneal pigmentations and deposits | 25265 | 25274 | 0 | 10 | nan [nan, nan] | 0.3148 | nan |
| 24m | Corneal pigmentations and deposits | 25226 | 25230 | 10 | 10 | 0.49 [0.04, 5.42] | 0.5537 | 0.0977 |
| 36m | Corneal pigmentations and deposits | 23890 | 23894 | 10 | 10 | 0.49 [0.09, 2.68] | 0.4018 | 0.7034 |
| 48m | Corneal pigmentations and deposits | 22607 | 22611 | 10 | 10 | 0.39 [0.08, 2.01] | 0.2419 | 0.781 |

| | | | | | | | | |
|---|---|---|---|---|---|---|---|---|
| 60m | Corneal pigmentations and deposits | 20898 | 20901 | 10 | 10 | 0.19 [0.02, 1.67] | 0.096 | 0.6434 |
| 120m | Corneal pigmentations and deposits | 22607 | 22611 | 10 | 10 | 0.33 [0.07, 1.63] | 0.1524 | 0.9673 |
| 6m | Bullous keratopathy | 25277 | 25268 | 10 | 0 | nan [nan, nan] | 0.3244 | nan |
| 12m | Bullous keratopathy | 25277 | 25268 | 10 | 0 | nan [nan, nan] | 0.1645 | nan |
| 24m | Bullous keratopathy | 25238 | 25229 | 10 | 0 | nan [nan, nan] | 0.1638 | nan |
| 36m | Bullous keratopathy | 23899 | 23892 | 10 | 10 | 0.97 [0.14, 6.88] | 0.9753 | 0.0733 |
| 48m | Bullous keratopathy | 22616 | 22609 | 10 | 10 | 0.39 [0.08, 2.0] | 0.2403 | 0.2 |
| 60m | Bullous keratopathy | 20904 | 20899 | 10 | 10 | 0.49 [0.04, 5.36] | 0.5469 | 0.2011 |
| 120m | Bullous keratopathy | 22616 | 22609 | 10 | 10 | 0.59 [0.14, 2.48] | 0.4688 | 0.9686 |
| 6m | Corneal degeneration | 25241 | 25222 | 10 | 10 | 0.43 [0.13, 1.41] | 0.1524 | 0.9469 |
| 12m | Corneal degeneration | 25241 | 25222 | 10 | 12 | 0.49 [0.18, 1.29] | 0.1401 | 0.8533 |
| 24m | Corneal degeneration | 25203 | 25190 | 10 | 12 | 0.65 [0.27, 1.59] | 0.3438 | 0.3323 |
| 36m | Corneal degeneration | 23870 | 23858 | 10 | 18 | 0.43 [0.19, 1.0] | 0.0426 | 0.5525 |
| 48m | Corneal degeneration | 22579 | 22569 | 13 | 19 | 0.66 [0.33, 1.34] | 0.2506 | 0.4402 |
| 60m | Corneal degeneration | 20871 | 20860 | 13 | 19 | 0.67 [0.33, 1.35] | 0.2541 | 0.5589 |
| 120m | Corneal degeneration | 22579 | 22569 | 18 | 23 | 0.78 [0.42, 1.45] | 0.4333 | 0.2067 |

| | | | | | | | | |
|---|---|---|---|---|---|---|---|---|
| 6m | Keratoconus | 25262 | 25258 | 10 | 10 | 0.33 [0.03, 3.13] | 0.3061 | 0.1768 |
| 12m | Keratoconus | 25262 | 25258 | 10 | 10 | 0.19 [0.02, 1.66] | 0.0954 | 0.1743 |
| 24m | Keratoconus | 25225 | 25217 | 10 | 10 | 0.78 [0.21, 2.91] | 0.7112 | 0.1185 |
| 36m | Keratoconus | 23885 | 23882 | 10 | 10 | 0.58 [0.14, 2.44] | 0.4541 | 0.3468 |
| 48m | Keratoconus | 22602 | 22598 | 10 | 10 | 0.39 [0.08, 1.99] | 0.2388 | 0.8883 |
| 60m | Keratoconus | 20890 | 20893 | 10 | 10 | 0.65 [0.18, 2.3] | 0.4981 | 0.3662 |
| 120m | Keratoconus | 22602 | 22598 | 10 | 10 | 0.85 [0.26, 2.79] | 0.7915 | 0.0477 |
| 6m | Iridocyclitis | 24966 | 25010 | 31 | 16 | 1.9 [1.04, 3.46] | 0.0347 | 0.5109 |
| 12m | Iridocyclitis | 24966 | 25010 | 44 | 28 | 1.53 [0.95, 2.46] | 0.0764 | 0.7054 |
| 24m | Iridocyclitis | 24924 | 24961 | 58 | 49 | 1.16 [0.79, 1.69] | 0.453 | 0.5925 |
| 36m | Iridocyclitis | 23609 | 23645 | 68 | 51 | 1.3 [0.91, 1.87] | 0.1527 | 0.3281 |
| 48m | Iridocyclitis | 22332 | 22374 | 77 | 57 | 1.31 [0.93, 1.85] | 0.1209 | 0.5807 |
| 60m | Iridocyclitis | 20653 | 20701 | 79 | 57 | 1.35 [0.96, 1.9] | 0.0841 | 0.8658 |
| 120m | Iridocyclitis | 22332 | 22374 | 91 | 73 | 1.23 [0.91, 1.68] | 0.18 | 0.7923 |
| 6m | Age-related cataract | 24416 | 24121 | 105 | 127 | 0.79 [0.61, 1.03] | 0.0799 | 0.9993 |
| 12m | Age-related cataract | 24416 | 24121 | 199 | 208 | 0.91 [0.75, 1.11] | 0.3525 | 0.0446 |

| | | | | | | | | |
|---|---|---|---|---|---|---|---|---|
| 24m | Age-related cataract | 24378 | 23966 | 336 | 357 | 0.9 [0.77, 1.04] | 0.1447 | 0.7032 |
| 36m | Age-related cataract | 23086 | 22824 | 413 | 438 | 0.9 [0.79, 1.03] | 0.127 | 0.7984 |
| 48m | Age-related cataract | 21866 | 21543 | 463 | 489 | 0.89 [0.79, 1.01] | 0.0807 | 0.9162 |
| 60m | Age-related cataract | 20259 | 19972 | 450 | 506 | 0.84 [0.74, 0.96] | 0.0076 | 0.7052 |
| 120m | Age-related cataract | 21866 | 21543 | 629 | 697 | 0.88 [0.79, 0.98] | 0.0188 | 0.8804 |
| 6m | Chorioretinal inflammation | 25236 | 25234 | 10 | 10 | 0.98 [0.2, 4.85] | 0.9787 | 0.9082 |
| 12m | Chorioretinal inflammation | 25236 | 25234 | 10 | 10 | 1.36 [0.43, 4.28] | 0.5994 | 0.7606 |
| 24m | Chorioretinal inflammation | 25195 | 25194 | 10 | 10 | 0.87 [0.33, 2.25] | 0.7701 | 0.2456 |
| 36m | Chorioretinal inflammation | 23863 | 23859 | 12 | 12 | 0.98 [0.44, 2.17] | 0.9528 | 0.2467 |
| 48m | Chorioretinal inflammation | 22578 | 22574 | 12 | 10 | 1.45 [0.59, 3.55] | 0.413 | 0.1113 |
| 60m | Chorioretinal inflammation | 20874 | 20872 | 12 | 11 | 1.06 [0.47, 2.4] | 0.8892 | 0.6548 |
| 120m | Chorioretinal inflammation | 22578 | 22574 | 16 | 12 | 1.32 [0.63, 2.8] | 0.4637 | 0.7307 |
| 6m | Other disorders of choroid | 25221 | 25196 | 10 | 10 | 0.56 [0.16, 1.9] | 0.3439 | 0.7434 |
| 12m | Other disorders of choroid | 25221 | 25196 | 10 | 10 | 0.68 [0.26, 1.78] | 0.4293 | 0.8572 |
| 24m | Other disorders of choroid | 25180 | 25149 | 11 | 16 | 0.67 [0.31, 1.44] | 0.3027 | 0.3595 |
| 36m | Other disorders of choroid | 23848 | 23827 | 18 | 16 | 1.1 [0.56, 2.15] | 0.7915 | 0.15 |

| | | | | | | | | |
|---|---|---|---|---|---|---|---|---|
| 48m | Other disorders of choroid | 22568 | 22544 | 22 | 15 | 1.42 [0.74, 2.73] | 0.2956 | 0.2245 |
| 60m | Other disorders of choroid | 20859 | 20844 | 24 | 17 | 1.37 [0.74, 2.55] | 0.3177 | 0.5606 |
| 120m | Other disorders of choroid | 22568 | 22544 | 31 | 23 | 1.35 [0.78, 2.31] | 0.2792 | 0.8599 |
| 6m | Retinal detachment with retinal break | 25245 | 25219 | 10 | 10 | 0.28 [0.06, 1.34] | 0.0873 | 0.2772 |
| 12m | Retinal detachment with retinal break | 25245 | 25219 | 10 | 10 | 0.48 [0.17, 1.42] | 0.1753 | 0.104 |
| 24m | Retinal detachment with retinal break | 25207 | 25175 | 10 | 13 | 0.6 [0.25, 1.45] | 0.2497 | 0.3117 |
| 36m | Retinal detachment with retinal break | 23872 | 23847 | 10 | 12 | 0.81 [0.35, 1.88] | 0.6226 | 0.0421 |
| 48m | Retinal detachment with retinal break | 22591 | 22558 | 15 | 13 | 1.11 [0.53, 2.34] | 0.7785 | 0.2839 |
| 60m | Retinal detachment with retinal break | 20881 | 20852 | 19 | 16 | 1.15 [0.59, 2.23] | 0.6816 | 0.037 |
| 120m | Retinal detachment with retinal break | 22591 | 22558 | 23 | 16 | 1.44 [0.76, 2.73] | 0.2603 | 0.1358 |
| 6m | Retinoschisis and retinal cysts | 25271 | 25258 | 0 | 10 | nan [nan, nan] | 0.3092 | nan |
| 12m | Retinoschisis and retinal cysts | 25271 | 25258 | 0 | 10 | nan [nan, nan] | 0.3092 | nan |
| 24m | Retinoschisis and retinal cysts | 25232 | 25219 | 10 | 10 | 0.97 [0.2, 4.83] | 0.9751 | 0.4894 |
| 36m | Retinoschisis and retinal cysts | 23896 | 23886 | 10 | 10 | 0.32 [0.03, 3.12] | 0.3044 | 0.5121 |
| 48m | Retinoschisis and retinal cysts | 22610 | 22601 | 10 | 10 | 0.72 [0.16, 3.22] | 0.6681 | 0.7982 |
| 60m | Retinoschisis and retinal cysts | 20898 | 20890 | 10 | 10 | 0.72 [0.16, 3.22] | 0.668 | 0.748 |

| | | | | | | | | |
|---|---|---|---|---|---|---|---|---|
| 120m | Retinoschisis and retinal cysts | 22610 | 22601 | 10 | 10 | 0.69 [0.19, 2.46] | 0.5666 | 0.3746 |
| 6m | Serous retinal detachment | 25249 | 25229 | 10 | 10 | 0.97 [0.24, 3.9] | 0.9705 | 0.4588 |
| 12m | Serous retinal detachment | 25249 | 25229 | 10 | 10 | 0.75 [0.28, 2.02] | 0.5718 | 0.2123 |
| 24m | Serous retinal detachment | 25210 | 25188 | 14 | 16 | 0.85 [0.42, 1.75] | 0.6653 | 0.7591 |
| 36m | Serous retinal detachment | 23877 | 23859 | 15 | 17 | 0.86 [0.43, 1.72] | 0.6715 | 0.9971 |
| 48m | Serous retinal detachment | 22591 | 22573 | 22 | 19 | 1.12 [0.61, 2.07] | 0.7182 | 0.4834 |
| 60m | Serous retinal detachment | 20883 | 20865 | 19 | 19 | 0.97 [0.51, 1.83] | 0.9251 | 0.26 |
| 120m | Serous retinal detachment | 22591 | 22573 | 27 | 25 | 1.06 [0.62, 1.83] | 0.824 | 0.8782 |
| 6m | Retinal breaks without detachment | 25235 | 25214 | 10 | 10 | 1.11 [0.4, 3.07] | 0.8352 | 0.6193 |
| 12m | Retinal breaks without detachment | 25235 | 25214 | 13 | 10 | 1.4 [0.6, 3.28] | 0.4339 | 0.5704 |
| 24m | Retinal breaks without detachment | 25196 | 25176 | 19 | 21 | 0.88 [0.47, 1.64] | 0.694 | 0.4045 |
| 36m | Retinal breaks without detachment | 23863 | 23845 | 19 | 22 | 0.84 [0.46, 1.56] | 0.5837 | 0.0648 |
| 48m | Retinal breaks without detachment | 22581 | 22558 | 25 | 28 | 0.87 [0.5, 1.48] | 0.598 | 0.3987 |
| 60m | Retinal breaks without detachment | 20874 | 20855 | 25 | 30 | 0.81 [0.48, 1.37] | 0.4293 | 0.6939 |
| 120m | Retinal breaks without detachment | 22581 | 22558 | 32 | 39 | 0.82 [0.52, 1.31] | 0.4119 | 0.9545 |
| 6m | Traction detachment of retina | 25272 | 25267 | 10 | 10 | 0.98 [0.14, 6.98] | 0.9865 | 0.0752 |

| | | | | | | | | |
|---|---|---|---|---|---|---|---|---|
| 12m | Traction detachment of retina | 25272 | 25267 | 10 | 10 | 0.98 [0.2, 4.84] | 0.9763 | 0.8933 |
| 24m | Traction detachment of retina | 25234 | 25227 | 10 | 10 | 0.98 [0.2, 4.85] | 0.9783 | 0.5315 |
| 36m | Traction detachment of retina | 23896 | 23893 | 10 | 10 | 1.22 [0.33, 4.55] | 0.7656 | 0.7652 |
| 48m | Traction detachment of retina | 22613 | 22606 | 10 | 10 | 0.97 [0.24, 3.86] | 0.9598 | 0.6806 |
| 60m | Traction detachment of retina | 20901 | 20893 | 10 | 10 | 1.21 [0.33, 4.52] | 0.7719 | 0.8849 |
| 120m | Traction detachment of retina | 22613 | 22606 | 10 | 10 | 0.71 [0.23, 2.25] | 0.564 | 0.3928 |
| 6m | Retinal vascular occlusions | 25221 | 25201 | 10 | 10 | 1.13 [0.38, 3.37] | 0.8226 | 0.3295 |
| 12m | Retinal vascular occlusions | 25221 | 25201 | 15 | 12 | 1.21 [0.57, 2.58] | 0.6264 | 0.4786 |
| 24m | Retinal vascular occlusions | 25179 | 25147 | 20 | 15 | 1.3 [0.66, 2.54] | 0.4439 | 0.3874 |
| 36m | Retinal vascular occlusions | 23847 | 23824 | 24 | 24 | 0.97 [0.55, 1.71] | 0.9213 | 0.7426 |
| 48m | Retinal vascular occlusions | 22560 | 22541 | 29 | 20 | 1.4 [0.79, 2.48] | 0.2434 | 0.0661 |
| 60m | Retinal vascular occlusions | 20854 | 20830 | 27 | 22 | 1.19 [0.68, 2.09] | 0.5445 | 0.843 |
| 120m | Retinal vascular occlusions | 22560 | 22541 | 39 | 41 | 0.97 [0.63, 1.5] | 0.8897 | 0.3691 |
| 6m | Transient retinal artery occlusion | 25267 | 25263 | 10 | 0 | nan [nan, nan] | 0.3241 | nan |
| 12m | Transient retinal artery occlusion | 25267 | 25263 | 10 | 0 | nan [nan, nan] | 0.1643 | nan |
| 24m | Transient retinal artery occlusion | 25227 | 25216 | 10 | 0 | nan [nan, nan] | 0.087 | nan |

| | | | | | | | | |
|---|---|---|---|---|---|---|---|---|
| 36m | Transient retinal artery occlusion | 23890 | 23884 | 10 | 10 | 1.96 [0.36, 10.69] | 0.4294 | 0.4708 |
| 48m | Transient retinal artery occlusion | 22605 | 22602 | 10 | 10 | 3.86 [0.43, 34.54] | 0.1928 | 0.5045 |
| 60m | Transient retinal artery occlusion | 20893 | 20886 | 10 | 10 | 1.3 [0.29, 5.8] | 0.7327 | 0.1448 |
| 120m | Transient retinal artery occlusion | 22605 | 22602 | 10 | 10 | 1.0 [0.25, 3.99] | 0.9971 | 0.0397 |
| 6m | Central retinal artery occlusion | 25272 | 25269 | 10 | 0 | nan [nan, nan] | 0.164 | nan |
| 12m | Central retinal artery occlusion | 25272 | 25269 | 10 | 10 | 2.9 [0.3, 27.84] | 0.3346 | 0.9332 |
| 24m | Central retinal artery occlusion | 25233 | 25227 | 10 | 10 | 1.95 [0.36, 10.67] | 0.4304 | 0.0534 |
| 36m | Central retinal artery occlusion | 23894 | 23892 | 10 | 10 | 1.94 [0.49, 7.78] | 0.3379 | 0.5801 |
| 48m | Central retinal artery occlusion | 22612 | 22610 | 10 | 10 | 1.16 [0.35, 3.8] | 0.8062 | 0.6869 |
| 60m | Central retinal artery occlusion | 20900 | 20898 | 10 | 10 | 1.16 [0.35, 3.8] | 0.8061 | 0.5764 |
| 120m | Central retinal artery occlusion | 22612 | 22610 | 10 | 10 | 0.9 [0.35, 2.33] | 0.8284 | 0.953 |
| 6m | Other retinal artery occlusions | 25265 | 25263 | 10 | 10 | 3.89 [0.44, 34.83] | 0.1897 | 0.0694 |
| 12m | Other retinal artery occlusions | 25265 | 25263 | 10 | 10 | 2.26 [0.58, 8.73] | 0.2252 | 0.7906 |
| 24m | Other retinal artery occlusions | 25225 | 25224 | 10 | 10 | 1.36 [0.43, 4.29] | 0.5955 | 0.4605 |
| 36m | Other retinal artery occlusions | 23889 | 23887 | 10 | 10 | 1.56 [0.51, 4.76] | 0.4342 | 0.1122 |
| 48m | Other retinal artery occlusions | 22605 | 22601 | 10 | 10 | 4.83 [1.06, 22.03] | 0.0245 | 0.8436 |

| | | | | | | | | |
|---|---|---|---|---|---|---|---|---|
| 60m | Other retinal artery occlusions | 20893 | 20890 | 11 | 10 | 3.55 [0.99, 12.73] | 0.0377 | 0.5192 |
| 120m | Other retinal artery occlusions | 22605 | 22601 | 14 | 10 | 3.53 [1.16, 10.74] | 0.0175 | 0.2335 |
| 6m | Other retinal vascular occlusions | 25244 | 25240 | 10 | 10 | 1.29 [0.29, 5.78] | 0.735 | 0.8635 |
| 12m | Other retinal vascular occlusions | 25244 | 25240 | 10 | 10 | 1.55 [0.51, 4.73] | 0.4408 | 0.4204 |
| 24m | Other retinal vascular occlusions | 25204 | 25197 | 13 | 10 | 2.53 [0.9, 7.11] | 0.0671 | 0.2901 |
| 36m | Other retinal vascular occlusions | 23867 | 23863 | 14 | 10 | 1.51 [0.65, 3.5] | 0.3292 | 0.8371 |
| 48m | Other retinal vascular occlusions | 22582 | 22579 | 18 | 10 | 1.74 [0.81, 3.78] | 0.1528 | 0.109 |
| 60m | Other retinal vascular occlusions | 20876 | 20865 | 15 | 10 | 1.82 [0.77, 4.3] | 0.1633 | 0.535 |
| 120m | Other retinal vascular occlusions | 22582 | 22579 | 23 | 25 | 0.95 [0.54, 1.68] | 0.863 | 0.2085 |
| 6m | Unspecified retinal vascular occlusion | 25273 | 25267 | 10 | 10 | 0.49 [0.04, 5.39] | 0.5502 | 0.1484 |
| 12m | Unspecified retinal vascular occlusion | 25273 | 25267 | 10 | 10 | 0.16 [0.02, 1.34] | 0.0533 | 0.3083 |
| 24m | Unspecified retinal vascular occlusion | 25234 | 25224 | 10 | 10 | 0.11 [0.01, 0.85] | 0.0101 | 0.1307 |
| 36m | Unspecified retinal vascular occlusion | 23896 | 23890 | 10 | 10 | 0.12 [0.02, 0.97] | 0.0173 | 0.1303 |
| 48m | Unspecified retinal vascular occlusion | 22613 | 22603 | 10 | 10 | 0.16 [0.02, 1.34] | 0.0529 | 0.1147 |
| 60m | Unspecified retinal vascular occlusion | 20902 | 20894 | 10 | 10 | 0.14 [0.02, 1.12] | 0.0296 | 0.1111 |
| 120m | Unspecified retinal vascular occlusion | 22613 | 22603 | 10 | 10 | 0.12 [0.02, 0.99] | 0.019 | 0.1283 |

| | | | | | | | | |
|---|---|---|---|---|---|---|---|---|
| 6m | Type 1 diabetes mellitus with ophthalmic complications | 25234 | 25221 | 10 | 10 | 1.29 [0.29, 5.78] | 0.7347 | 0.4732 |
| 12m | Type 1 diabetes mellitus with ophthalmic complications | 25234 | 25221 | 10 | 10 | 1.29 [0.29, 5.78] | 0.7347 | 0.6465 |
| 24m | Type 1 diabetes mellitus with ophthalmic complications | 25198 | 25182 | 10 | 10 | 0.84 [0.28, 2.49] | 0.7468 | 0.9078 |
| 36m | Type 1 diabetes mellitus with ophthalmic complications | 23863 | 23851 | 10 | 10 | 1.09 [0.44, 2.67] | 0.8584 | 0.93 |
| 48m | Type 1 diabetes mellitus with ophthalmic complications | 22577 | 22567 | 10 | 10 | 0.97 [0.4, 2.34] | 0.95 | 0.464 |
| 60m | Type 1 diabetes mellitus with ophthalmic complications | 20868 | 20856 | 12 | 11 | 1.06 [0.47, 2.41] | 0.8821 | 0.3045 |
| 120m | Type 1 diabetes mellitus with ophthalmic complications | 22577 | 22567 | 16 | 16 | 1.01 [0.5, 2.02] | 0.9785 | 0.705 |
| 6m | Type 2 diabetes mellitus with ophthalmic complications | 24989 | 24960 | 40 | 51 | 0.76 [0.51, 1.16] | 0.2011 | 0.3989 |
| 12m | Type 2 diabetes mellitus with ophthalmic complications | 24989 | 24960 | 57 | 76 | 0.73 [0.52, 1.03] | 0.0679 | 0.6667 |
| 24m | Type 2 diabetes mellitus with ophthalmic complications | 24954 | 24909 | 100 | 98 | 0.99 [0.75, 1.31] | 0.9657 | 0.0729 |

| | | | | | | | | |
|---|---|---|---|---|---|---|---|---|
| 36m | Type 2 diabetes mellitus with ophthalmic complications | 23636 | 23618 | 127 | 114 | 1.09 [0.84, 1.4] | 0.5197 | 0.0656 |
| 48m | Type 2 diabetes mellitus with ophthalmic complications | 22361 | 22318 | 151 | 154 | 0.95 [0.76, 1.19] | 0.6372 | 0.3866 |
| 60m | Type 2 diabetes mellitus with ophthalmic complications | 20680 | 20634 | 147 | 156 | 0.91 [0.73, 1.14] | 0.4315 | 0.6757 |
| 120m | Type 2 diabetes mellitus with ophthalmic complications | 22361 | 22318 | 233 | 224 | 1.05 [0.87, 1.26] | 0.6191 | 0.0458 |
| 6m | Other specified diabetes mellitus with ophthalmic complications | 25270 | 25269 | 10 | 10 | 2.93 [0.3, 28.15] | 0.3291 | 0.5242 |
| 12m | Other specified diabetes mellitus with ophthalmic complications | 25270 | 25269 | 10 | 10 | 1.94 [0.36, 10.61] | 0.4346 | 0.9157 |
| 24m | Other specified diabetes mellitus with ophthalmic complications | 25231 | 25224 | 10 | 10 | 1.17 [0.36, 3.84] | 0.7922 | 0.7758 |
| 36m | Other specified diabetes mellitus with ophthalmic complications | 23893 | 23891 | 10 | 10 | 0.86 [0.31, 2.36] | 0.7648 | 0.6989 |
| 48m | Other specified diabetes mellitus with ophthalmic complications | 22610 | 22607 | 10 | 10 | 1.29 [0.45, 3.73] | 0.6323 | 0.7899 |
| 60m | Other specified diabetes mellitus with ophthalmic complications | 20899 | 20894 | 10 | 10 | 1.56 [0.51, 4.75] | 0.435 | 0.0891 |

| | | | | | | | | |
|---|---|---|---|---|---|---|---|---|
| 120m | Other specified diabetes mellitus with ophthalmic complications | 22610 | 22607 | 10 | 10 | 1.12 [0.43, 2.91] | 0.8139 | 0.7286 |
| 6m | Background retinopathy and retinal vascular changes | 25194 | 25144 | 10 | 12 | 0.57 [0.22, 1.44] | 0.2289 | 0.663 |
| 12m | Background retinopathy and retinal vascular changes | 25194 | 25144 | 15 | 17 | 0.85 [0.43, 1.71] | 0.6572 | 0.3814 |
| 24m | Background retinopathy and retinal vascular changes | 25155 | 25093 | 37 | 36 | 1.0 [0.63, 1.58] | 0.9977 | 0.0823 |
| 36m | Background retinopathy and retinal vascular changes | 23823 | 23779 | 43 | 34 | 1.23 [0.79, 1.93] | 0.3619 | 0.8819 |
| 48m | Background retinopathy and retinal vascular changes | 22543 | 22495 | 52 | 49 | 1.03 [0.69, 1.51] | 0.8998 | 0.9351 |
| 60m | Background retinopathy and retinal vascular changes | 20836 | 20792 | 47 | 53 | 0.86 [0.58, 1.27] | 0.4522 | 0.3638 |
| 120m | Background retinopathy and retinal vascular changes | 22543 | 22495 | 67 | 81 | 0.84 [0.6, 1.16] | 0.279 | 0.0483 |
| 6m | Other retinal detachments | 25270 | 25265 | 10 | 10 | 0.98 [0.06, 15.66] | 0.9882 | 0.1562 |
| 12m | Other retinal detachments | 25270 | 25265 | 10 | 10 | 2.91 [0.3, 28.01] | 0.3317 | 0.5341 |
| 24m | Other retinal detachments | 25232 | 25226 | 10 | 10 | 1.47 [0.24, 8.77] | 0.6735 | 0.0521 |
| 36m | Other retinal detachments | 23894 | 23892 | 10 | 10 | 0.65 [0.11, 3.91] | 0.6391 | 0.417 |
| 48m | Other retinal detachments | 22610 | 22606 | 10 | 10 | 0.77 [0.21, 2.88] | 0.7014 | 0.1604 |
| 60m | Other retinal detachments | 20898 | 20892 | 10 | 10 | 1.21 [0.33, 4.52] | 0.773 | 0.9152 |
| 120m | Other retinal detachments | 22610 | 22606 | 10 | 10 | 1.18 [0.36, 3.87] | 0.7831 | 0.9772 |

| | | | | | | | | |
|---|---|---|---|---|---|---|---|---|
| 6m | AMD | 24997 | 24842 | 38 | 55 | 0.67 [0.44, 1.01] | 0.055 | 0.375 |
| 12m | AMD | 24997 | 24842 | 63 | 92 | 0.66 [0.48, 0.91] | 0.0102 | 0.5198 |
| 24m | AMD | 24956 | 24788 | 105 | 150 | 0.68 [0.53, 0.87] | 0.002 | 0.3113 |
| 36m | AMD | 23643 | 23505 | 135 | 192 | 0.68 [0.54, 0.85] | 0.0005 | 0.6744 |
| 48m | AMD | 22369 | 22213 | 149 | 209 | 0.68 [0.55, 0.84] | 0.0003 | 0.3852 |
| 60m | AMD | 20686 | 20549 | 148 | 211 | 0.67 [0.55, 0.83] | 0.0002 | 0.8018 |
| 120m | AMD | 22369 | 22213 | 220 | 315 | 0.7 [0.59, 0.83] | <0.0001 | 0.5659 |
| 6m | nvAMD | 25255 | 25245 | 10 | 10 | 0.97 [0.2, 4.81] | 0.9709 | 0.6181 |
| 12m | nvAMD | 25255 | 25245 | 10 | 12 | 0.4 [0.14, 1.14] | 0.0764 | 0.2499 |
| 24m | nvAMD | 25216 | 25207 | 10 | 11 | 0.79 [0.33, 1.92] | 0.608 | 0.9741 |
| 36m | nvAMD | 23877 | 23877 | 12 | 25 | 0.47 [0.23, 0.93] | 0.0265 | 0.4001 |
| 48m | nvAMD | 22596 | 22586 | 10 | 15 | 0.58 [0.25, 1.32] | 0.1879 | 0.8386 |
| 60m | nvAMD | 20885 | 20869 | 10 | 18 | 0.48 [0.22, 1.08] | 0.0701 | 0.751 |
| 120m | nvAMD | 22596 | 22586 | 18 | 30 | 0.63 [0.35, 1.12] | 0.1121 | 0.5303 |
| 6m | non-nvAMD | 25216 | 25170 | 10 | 10 | 0.97 [0.31, 3.01] | 0.9606 | 0.1789 |
| 12m | non-nvAMD | 25216 | 25170 | 13 | 16 | 0.78 [0.38, 1.63] | 0.5137 | 0.4943 |

| | | | | | | | | |
|---|---|---|---|---|---|---|---|---|
| 24m | non-nvAMD | 25177 | 25139 | 21 | 29 | 0.7 [0.4, 1.23] | 0.2158 | 0.6937 |
| 36m | non-nvAMD | 23842 | 23804 | 23 | 42 | 0.53 [0.32, 0.89] | 0.0135 | 0.2464 |
| 48m | non-nvAMD | 22558 | 22520 | 29 | 44 | 0.63 [0.4, 1.01] | 0.0553 | 0.5664 |
| 60m | non-nvAMD | 20854 | 20817 | 35 | 51 | 0.66 [0.43, 1.02] | 0.0607 | 0.3427 |
| 120m | non-nvAMD | 22558 | 22520 | 50 | 69 | 0.73 [0.51, 1.05] | 0.0895 | 0.9476 |
| 6m | Glaucoma | 24629 | 24533 | 66 | 86 | 0.74 [0.54, 1.03] | 0.0708 | 0.8797 |
| 12m | Glaucoma | 24629 | 24533 | 104 | 128 | 0.78 [0.61, 1.02] | 0.0647 | 0.5527 |
| 24m | Glaucoma | 24599 | 24466 | 152 | 186 | 0.79 [0.64, 0.98] | 0.0308 | 0.6235 |
| 36m | Glaucoma | 23290 | 23189 | 176 | 227 | 0.75 [0.61, 0.91] | 0.0038 | 0.6525 |
| 48m | Glaucoma | 22051 | 21951 | 199 | 249 | 0.77 [0.64, 0.92] | 0.0049 | 0.7393 |
| 60m | Glaucoma | 20425 | 20307 | 194 | 246 | 0.76 [0.63, 0.91] | 0.0035 | 0.662 |
| 120m | Glaucoma | 22051 | 21951 | 259 | 351 | 0.73 [0.62, 0.86] | 0.0001 | 0.7686 |
| 6m | Glaucoma suspect | 24871 | 24791 | 45 | 53 | 0.82 [0.55, 1.23] | 0.3402 | 0.7356 |
| 12m | Glaucoma suspect | 24871 | 24791 | 75 | 82 | 0.88 [0.65, 1.21] | 0.4371 | 0.524 |
| 24m | Glaucoma suspect | 24841 | 24726 | 114 | 124 | 0.89 [0.69, 1.15] | 0.3701 | 0.5831 |
| 36m | Glaucoma suspect | 23514 | 23435 | 133 | 145 | 0.89 [0.7, 1.12] | 0.3202 | 0.9235 |

| | | | | | | | | |
|---|---|---|---|---|---|---|---|---|
| 48m | Glaucoma suspect | 22261 | 22178 | 153 | 174 | 0.84 [0.68, 1.05] | 0.1261 | 0.3009 |
| 60m | Glaucoma suspect | 20612 | 20518 | 146 | 162 | 0.87 [0.69, 1.08] | 0.2113 | 0.865 |
| 120m | Glaucoma suspect | 22261 | 22178 | 197 | 239 | 0.81 [0.67, 0.98] | 0.0303 | 0.5418 |
| 6m | Open-angle glaucoma | 25147 | 25122 | 14 | 19 | 0.72 [0.36, 1.43] | 0.3414 | 0.5287 |
| 12m | Open-angle glaucoma | 25147 | 25122 | 22 | 24 | 0.89 [0.5, 1.58] | 0.6877 | 0.2883 |
| 24m | Open-angle glaucoma | 25108 | 25088 | 34 | 32 | 1.04 [0.64, 1.68] | 0.8879 | 0.6571 |
| 36m | Open-angle glaucoma | 23776 | 23760 | 40 | 50 | 0.78 [0.51, 1.18] | 0.2374 | 0.2148 |
| 48m | Open-angle glaucoma | 22506 | 22477 | 41 | 44 | 0.9 [0.59, 1.38] | 0.6296 | 0.7775 |
| 60m | Open-angle glaucoma | 20815 | 20788 | 38 | 53 | 0.69 [0.46, 1.05] | 0.0849 | 0.4564 |
| 120m | Open-angle glaucoma | 22506 | 22477 | 63 | 86 | 0.75 [0.54, 1.03] | 0.0783 | 0.6183 |
| 6m | Primary angle-closure glaucoma | 25254 | 25253 | 10 | 10 | 1.47 [0.25, 8.81] | 0.6703 | 0.9465 |
| 12m | Primary angle-closure glaucoma | 25254 | 25253 | 10 | 10 | 0.97 [0.28, 3.36] | 0.9637 | 0.397 |
| 24m | Primary angle-closure glaucoma | 25215 | 25213 | 10 | 10 | 0.68 [0.26, 1.79] | 0.4359 | 0.9397 |
| 36m | Primary angle-closure glaucoma | 23876 | 23880 | 10 | 11 | 0.53 [0.2, 1.44] | 0.2076 | 0.6407 |
| 48m | Primary angle-closure glaucoma | 22594 | 22598 | 10 | 10 | 0.86 [0.33, 2.23] | 0.7592 | 0.459 |
| 60m | Primary angle-closure glaucoma | 20884 | 20883 | 10 | 10 | 0.87 [0.36, 2.15] | 0.7711 | 0.4672 |

| | | | | | | | | |
|---|---|---|---|---|---|---|---|---|
| 120m | Primary angle-closure glaucoma | 22594 | 22598 | 11 | 14 | 0.8 [0.36, 1.75] | 0.5687 | 0.2091 |
| 6m | Glaucoma secondary to eye trauma | 25273 | 25274 | 0 | 0 | nan [nan, nan] | 1 | nan |
| 12m | Glaucoma secondary to eye trauma | 25273 | 25274 | 0 | 0 | nan [nan, nan] | 1 | nan |
| 24m | Glaucoma secondary to eye trauma | 25235 | 25236 | 0 | 0 | nan [nan, nan] | 1 | nan |
| 36m | Glaucoma secondary to eye trauma | 23896 | 23898 | 0 | 10 | nan [nan, nan] | 0.3177 | nan |
| 48m | Glaucoma secondary to eye trauma | 22614 | 22614 | 0 | 0 | nan [nan, nan] | 1 | nan |
| 60m | Glaucoma secondary to eye trauma | 20902 | 20902 | 0 | 0 | nan [nan, nan] | 1 | nan |
| 120m | Glaucoma secondary to eye trauma | 22614 | 22614 | 0 | 10 | nan [nan, nan] | 0.3451 | nan |
| 6m | Glaucoma secondary to eye inflammation | 25260 | 25262 | 10 | 10 | 1.29 [0.29, 5.78] | 0.735 | 0.4805 |
| 12m | Glaucoma secondary to eye inflammation | 25260 | 25262 | 10 | 10 | 0.97 [0.24, 3.88] | 0.9652 | 0.1964 |
| 24m | Glaucoma secondary to eye inflammation | 25221 | 25222 | 10 | 10 | 0.97 [0.24, 3.9] | 0.9709 | 0.1961 |
| 36m | Glaucoma secondary to eye inflammation | 23884 | 23890 | 10 | 10 | 0.43 [0.13, 1.41] | 0.1529 | 0.0971 |
| 48m | Glaucoma secondary to eye inflammation | 22601 | 22606 | 10 | 10 | 0.48 [0.17, 1.42] | 0.1757 | 0.9774 |
| 60m | Glaucoma secondary to eye inflammation | 20891 | 20892 | 10 | 10 | 0.97 [0.28, 3.35] | 0.9632 | 0.8354 |
| 120m | Glaucoma secondary to eye inflammation | 22601 | 22606 | 10 | 11 | 0.53 [0.2, 1.44] | 0.2044 | 0.9453 |
| 6m | Glaucoma secondary to other eye disorders | 25255 | 25252 | 10 | 10 | 1.46 [0.24, 8.72] | 0.6782 | 0.2383 |

| | | | | | | | | |
|---|---|---|---|---|---|---|---|---|
| 12m | Glaucoma secondary to other eye disorders | 25255 | 25252 | 10 | 10 | 1.46 [0.24, 8.72] | 0.6782 | 0.4479 |
| 24m | Glaucoma secondary to other eye disorders | 25216 | 25216 | 10 | 10 | 1.96 [0.49, 7.83] | 0.333 | 0.98 |
| 36m | Glaucoma secondary to other eye disorders | 23878 | 23881 | 10 | 10 | 1.37 [0.44, 4.32] | 0.5883 | 0.8329 |
| 48m | Glaucoma secondary to other eye disorders | 22597 | 22595 | 10 | 10 | 2.59 [0.69, 9.76] | 0.1444 | 0.2126 |
| 60m | Glaucoma secondary to other eye disorders | 20890 | 20887 | 10 | 10 | 7.78 [0.97, 62.21] | 0.0219 | 0.7963 |
| 120m | Glaucoma secondary to other eye disorders | 22597 | 22595 | 11 | 10 | 2.3 [0.8, 6.64] | 0.1118 | 0.3559 |
| 6m | Glaucoma secondary to drugs | 25267 | 25273 | 10 | 10 | 0.97 [0.06, 15.53] | 0.9835 | 0.1577 |
| 12m | Glaucoma secondary to drugs | 25267 | 25273 | 10 | 10 | 0.48 [0.04, 5.34] | 0.5447 | 0.9667 |
| 24m | Glaucoma secondary to drugs | 25230 | 25234 | 10 | 10 | 0.49 [0.04, 5.35] | 0.5463 | 0.9853 |
| 36m | Glaucoma secondary to drugs | 23892 | 23897 | 10 | 10 | 0.32 [0.03, 3.12] | 0.3038 | 0.5851 |
| 48m | Glaucoma secondary to drugs | 22612 | 22614 | 10 | 10 | 0.64 [0.11, 3.85] | 0.6268 | 0.3419 |
| 60m | Glaucoma secondary to drugs | 20899 | 20900 | 10 | 10 | 1.94 [0.18, 21.42] | 0.581 | 0.7282 |
| 120m | Glaucoma secondary to drugs | 22612 | 22614 | 10 | 10 | 0.52 [0.13, 2.07] | 0.3441 | 0.4811 |
| 6m | Other glaucoma | 25256 | 25260 | 0 | 10 | nan [nan, nan] | 0.0434 | nan |
| 12m | Other glaucoma | 25256 | 25260 | 0 | 10 | nan [nan, nan] | 0.0237 | nan |
| 24m | Other glaucoma | 25218 | 25225 | 10 | 10 | 0.82 [0.25, 2.67] | 0.7355 | 0.0346 |

| | | | | | | | | |
|---|---|---|---|---|---|---|---|---|
| 36m | Other glaucoma | 23881 | 23888 | 10 | 10 | 1.11 [0.43, 2.86] | 0.8367 | 0.0956 |
| 48m | Other glaucoma | 22599 | 22605 | 13 | 11 | 1.15 [0.52, 2.57] | 0.7329 | 0.0163 |
| 60m | Other glaucoma | 20889 | 20893 | 10 | 12 | 0.73 [0.31, 1.74] | 0.4774 | 0.7351 |
| 120m | Other glaucoma | 22599 | 22605 | 19 | 15 | 1.28 [0.65, 2.52] | 0.4746 | 0.1027 |
| 6m | Unspecified glaucoma | 25033 | 24997 | 25 | 32 | 0.76 [0.45, 1.28] | 0.3047 | 0.758 |
| 12m | Unspecified glaucoma | 25033 | 24997 | 36 | 51 | 0.68 [0.45, 1.05] | 0.079 | 0.2553 |
| 24m | Unspecified glaucoma | 24996 | 24966 | 52 | 72 | 0.7 [0.49, 1.0] | 0.0509 | 0.4868 |
| 36m | Unspecified glaucoma | 23677 | 23651 | 56 | 98 | 0.56 [0.4, 0.77] | 0.0004 | 0.1398 |
| 48m | Unspecified glaucoma | 22411 | 22381 | 59 | 110 | 0.52 [0.38, 0.71] | <0.0001 | 0.3213 |
| 60m | Unspecified glaucoma | 20715 | 20702 | 56 | 116 | 0.47 [0.34, 0.64] | <0.0001 | 0.5841 |
| 120m | Unspecified glaucoma | 22411 | 22381 | 79 | 166 | 0.48 [0.36, 0.62] | <0.0001 | 0.7621 |
| 6m | Disorders of vitreous body | 24881 | 24678 | 50 | 73 | 0.66 [0.46, 0.95] | 0.0232 | 0.0945 |
| 12m | Disorders of vitreous body | 24881 | 24678 | 100 | 130 | 0.74 [0.57, 0.96] | 0.0212 | 0.4554 |
| 24m | Disorders of vitreous body | 24841 | 24577 | 163 | 221 | 0.71 [0.58, 0.87] | 0.0008 | 0.8612 |
| 36m | Disorders of vitreous body | 23528 | 23348 | 207 | 265 | 0.75 [0.63, 0.9] | 0.0019 | 0.1016 |
| 48m | Disorders of vitreous body | 22257 | 22030 | 236 | 305 | 0.73 [0.62, 0.87] | 0.0003 | 0.2853 |

| | | | | | | | | |
|---|---|---|---|---|---|---|---|---|
| 60m | Disorders of vitreous body | 20608 | 20396 | 241 | 281 | 0.82 [0.69, 0.97] | 0.0206 | 0.1733 |
| 120m | Disorders of vitreous body | 22257 | 22030 | 335 | 437 | 0.75 [0.65, 0.87] | 0.0001 | 0.3757 |
| 6m | Disorders of globe | 25197 | 25194 | 10 | 11 | 0.53 [0.2, 1.44] | 0.2068 | 0.8807 |
| 12m | Disorders of globe | 25197 | 25194 | 10 | 12 | 0.73 [0.31, 1.73] | 0.4738 | 0.0822 |
| 24m | Disorders of globe | 25158 | 25153 | 15 | 26 | 0.56 [0.3, 1.06] | 0.0717 | 0.5856 |
| 36m | Disorders of globe | 23829 | 23827 | 20 | 31 | 0.63 [0.36, 1.11] | 0.1046 | 0.8361 |
| 48m | Disorders of globe | 22541 | 22543 | 25 | 37 | 0.65 [0.39, 1.09] | 0.0993 | 0.9947 |
| 60m | Disorders of globe | 20839 | 20834 | 25 | 42 | 0.58 [0.35, 0.95] | 0.0278 | 0.5229 |
| 120m | Disorders of globe | 22541 | 22543 | 35 | 43 | 0.81 [0.52, 1.26] | 0.3447 | 0.1729 |
| 6m | Optic neuritis | 25229 | 25193 | 10 | 10 | 0.81 [0.25, 2.66] | 0.7302 | 0.9042 |
| 12m | Optic neuritis | 25229 | 25193 | 10 | 10 | 0.54 [0.18, 1.61] | 0.2611 | 0.2223 |
| 24m | Optic neuritis | 25191 | 25148 | 10 | 15 | 0.58 [0.26, 1.33] | 0.1968 | 0.4339 |
| 36m | Optic neuritis | 23859 | 23818 | 13 | 18 | 0.7 [0.34, 1.44] | 0.3314 | 0.4517 |
| 48m | Optic neuritis | 22573 | 22533 | 16 | 22 | 0.7 [0.37, 1.34] | 0.2833 | 0.6491 |
| 60m | Optic neuritis | 20866 | 20829 | 18 | 25 | 0.7 [0.38, 1.28] | 0.2474 | 0.159 |
| 120m | Optic neuritis | 22573 | 22533 | 21 | 32 | 0.65 [0.37, 1.13] | 0.1222 | 0.7488 |

| | | | | | | | | |
|---|---|---|---|---|---|---|---|---|
| 6m | Ischemic optic neuropathy | 25262 | 25260 | 10 | 10 | 0.48 [0.04, 5.34] | 0.5447 | 0.2409 |
| 12m | Ischemic optic neuropathy | 25262 | 25260 | 10 | 10 | 0.8 [0.25, 2.63] | 0.7163 | 0.8962 |
| 24m | Ischemic optic neuropathy | 25223 | 25220 | 10 | 10 | 0.81 [0.25, 2.65] | 0.7254 | 0.3707 |
| 36m | Ischemic optic neuropathy | 23888 | 23884 | 10 | 10 | 0.48 [0.17, 1.41] | 0.1751 | 0.3387 |
| 48m | Ischemic optic neuropathy | 22603 | 22604 | 10 | 10 | 0.69 [0.22, 2.16] | 0.5166 | 0.241 |
| 60m | Ischemic optic neuropathy | 20893 | 20888 | 10 | 10 | 0.54 [0.18, 1.6] | 0.2572 | 0.0589 |
| 120m | Ischemic optic neuropathy | 22603 | 22604 | 10 | 10 | 0.81 [0.32, 2.05] | 0.6547 | 0.8559 |
| 6m | Optic atrophy | 25210 | 25180 | 10 | 10 | 0.39 [0.12, 1.25] | 0.0995 | 0.2624 |
| 12m | Optic atrophy | 25210 | 25180 | 10 | 19 | 0.41 [0.18, 0.93] | 0.0279 | 0.8902 |
| 24m | Optic atrophy | 25168 | 25131 | 18 | 33 | 0.53 [0.3, 0.94] | 0.0282 | 0.6416 |
| 36m | Optic atrophy | 23837 | 23808 | 15 | 33 | 0.44 [0.24, 0.81] | 0.0071 | 0.9755 |
| 48m | Optic atrophy | 22553 | 22524 | 20 | 42 | 0.46 [0.27, 0.78] | 0.0034 | 0.9581 |
| 60m | Optic atrophy | 20854 | 20814 | 21 | 47 | 0.43 [0.26, 0.73] | 0.0011 | 0.2203 |
| 120m | Optic atrophy | 22553 | 22524 | 37 | 60 | 0.63 [0.42, 0.94] | 0.0236 | 0.2057 |
| 6m | Paralytic strabismus | 25236 | 25225 | 10 | 10 | 0.7 [0.22, 2.2] | 0.5376 | 0.1111 |
| 12m | Paralytic strabismus | 25236 | 25225 | 11 | 12 | 0.89 [0.39, 2.02] | 0.7783 | 0.6315 |

| | | | | | | | | |
|---|---|---|---|---|---|---|---|---|
| 24m | Paralytic strabismus | 25197 | 25176 | 13 | 16 | 0.79 [0.38, 1.64] | 0.5286 | 0.3312 |
| 36m | Paralytic strabismus | 23864 | 23847 | 17 | 25 | 0.66 [0.36, 1.23] | 0.1882 | 0.6132 |
| 48m | Paralytic strabismus | 22581 | 22561 | 20 | 26 | 0.74 [0.41, 1.33] | 0.3168 | 0.8319 |
| 60m | Paralytic strabismus | 20870 | 20854 | 18 | 30 | 0.58 [0.32, 1.04] | 0.0644 | 0.0565 |
| 120m | Paralytic strabismus | 22581 | 22561 | 24 | 35 | 0.68 [0.41, 1.15] | 0.146 | 0.463 |
| 6m | Third oculomotor nerve palsy | 25268 | 25264 | 10 | 10 | 0.49 [0.04, 5.44] | 0.5561 | 0.9704 |
| 12m | Third oculomotor nerve palsy | 25268 | 25264 | 10 | 10 | 0.65 [0.11, 3.9] | 0.6361 | 0.4132 |
| 24m | Third oculomotor nerve palsy | 25229 | 25220 | 10 | 10 | 0.98 [0.24, 3.92] | 0.9768 | 0.2208 |
| 36m | Third oculomotor nerve palsy | 23891 | 23887 | 10 | 10 | 0.7 [0.22, 2.2] | 0.5348 | 0.8669 |
| 48m | Third oculomotor nerve palsy | 22607 | 22600 | 10 | 10 | 1.13 [0.38, 3.36] | 0.8281 | 0.3941 |
| 60m | Third oculomotor nerve palsy | 20895 | 20889 | 10 | 10 | 0.97 [0.34, 2.77] | 0.9551 | 0.4721 |
| 120m | Third oculomotor nerve palsy | 22607 | 22600 | 10 | 12 | 0.77 [0.32, 1.83] | 0.5525 | 0.3376 |
| 6m | Dry eye syndrome | 24743 | 23942 | 68 | 128 | 0.5 [0.37, 0.67] | <0.0001 | 0.8438 |
| 12m | Dry eye syndrome | 24743 | 23942 | 118 | 231 | 0.48 [0.38, 0.59] | <0.0001 | 0.9649 |
| 24m | Dry eye syndrome | 24713 | 23872 | 199 | 348 | 0.53 [0.45, 0.63] | <0.0001 | 0.2245 |
| 36m | Dry eye syndrome | 23411 | 22667 | 253 | 388 | 0.61 [0.52, 0.71] | <0.0001 | 0.0084 |

| | | | | | | | | |
|---|---|---|---|---|---|---|---|---|
| 48m | Dry eye syndrome | 22165 | 21418 | 277 | 441 | 0.58 [0.5, 0.67] | <0.0001 | 0.1342 |
| 60m | Dry eye syndrome | 20525 | 19876 | 272 | 430 | 0.58 [0.5, 0.68] | <0.0001 | 0.6829 |
| 120m | Dry eye syndrome | 22165 | 21418 | 378 | 606 | 0.59 [0.52, 0.67] | <0.0001 | 0.1725 |
| 6m | Diplopia | 25141 | 25069 | 22 | 15 | 1.43 [0.74, 2.75] | 0.2856 | 0.9208 |
| 12m | Diplopia | 25141 | 25069 | 31 | 37 | 0.81 [0.5, 1.31] | 0.3856 | 0.0732 |
| 24m | Diplopia | 25095 | 25029 | 46 | 70 | 0.64 [0.44, 0.93] | 0.0173 | 0.2895 |
| 36m | Diplopia | 23777 | 23713 | 62 | 77 | 0.78 [0.56, 1.1] | 0.1529 | 0.9993 |
| 48m | Diplopia | 22483 | 22414 | 65 | 97 | 0.65 [0.47, 0.88] | 0.006 | 0.7893 |
| 60m | Diplopia | 20781 | 20719 | 66 | 96 | 0.67 [0.49, 0.91] | 0.0105 | 0.2101 |
| 120m | Diplopia | 22483 | 22414 | 80 | 135 | 0.59 [0.44, 0.77] | 0.0001 | 0.263 |
| 6m | Visual disturbances | 24346 | 23992 | 119 | 149 | 0.77 [0.6, 0.97] | 0.0289 | 0.7918 |
| 12m | Visual disturbances | 24346 | 23992 | 204 | 256 | 0.76 [0.63, 0.91] | 0.003 | 0.5753 |
| 24m | Visual disturbances | 24288 | 23910 | 320 | 400 | 0.76 [0.66, 0.88] | 0.0003 | 0.9765 |
| 36m | Visual disturbances | 23064 | 22782 | 371 | 469 | 0.76 [0.66, 0.87] | 0.0001 | 0.925 |
| 48m | Visual disturbances | 21774 | 21407 | 400 | 510 | 0.74 [0.65, 0.84] | <0.0001 | 0.7643 |
| 60m | Visual disturbances | 20129 | 19789 | 420 | 515 | 0.77 [0.68, 0.88] | 0.0001 | 0.7315 |

| | | | | | | | | |
|---|---|---|---|---|---|---|---|---|
| 120m | Visual disturbances | 21774 | 21407 | 537 | 683 | 0.76 [0.68, 0.85] | <0.0001 | 0.5313 |
| 6m | Blindness and low vision | 24995 | 24903 | 37 | 48 | 0.75 [0.49, 1.15] | 0.1835 | 0.7144 |
| 12m | Blindness and low vision | 24995 | 24903 | 54 | 78 | 0.67 [0.47, 0.95] | 0.022 | 0.4919 |
| 24m | Blindness and low vision | 24945 | 24865 | 88 | 102 | 0.84 [0.63, 1.11] | 0.2196 | 0.3978 |
| 36m | Blindness and low vision | 23641 | 23586 | 99 | 121 | 0.79 [0.61, 1.04] | 0.0876 | 0.0162 |
| 48m | Blindness and low vision | 22363 | 22300 | 110 | 121 | 0.87 [0.68, 1.13] | 0.3086 | 0.5206 |
| 60m | Blindness and low vision | 20662 | 20602 | 108 | 139 | 0.75 [0.58, 0.96] | 0.0246 | 0.8265 |
| 120m | Blindness and low vision | 22363 | 22300 | 152 | 178 | 0.85 [0.69, 1.06] | 0.1436 | 0.4222 |
| 6m | Intraoperative and postprocedural complications and disorders of eye and adnexa, not elsewhere classified | 25171 | 25143 | 10 | 10 | 0.87 [0.34, 2.25] | 0.7717 | 0.9187 |
| 12m | Intraoperative and postprocedural complications and disorders of eye and adnexa, not elsewhere classified | 25171 | 25143 | 14 | 18 | 0.75 [0.37, 1.52] | 0.4263 | 0.8278 |
| 24m | Intraoperative and postprocedural complications and disorders of eye and adnexa, not elsewhere classified | 25129 | 25109 | 20 | 25 | 0.78 [0.43, 1.4] | 0.4053 | 0.3659 |

| | | | | | | | | |
|---|---|---|---|---|---|---|---|---|
| 36m | Intraoperative and postprocedural complications and disorders of eye and adnexa, not elsewhere classified | 23805 | 23785 | 23 | 30 | 0.75 [0.43, 1.28] | 0.2892 | 0.982 |
| 48m | Intraoperative and postprocedural complications and disorders of eye and adnexa, not elsewhere classified | 22519 | 22497 | 28 | 33 | 0.82 [0.5, 1.36] | 0.4408 | 0.4061 |
| 60m | Intraoperative and postprocedural complications and disorders of eye and adnexa, not elsewhere classified | 20808 | 20789 | 27 | 31 | 0.84 [0.5, 1.41] | 0.517 | 0.833 |
| 120m | Intraoperative and postprocedural complications and disorders of eye and adnexa, not elsewhere classified | 22519 | 22497 | 29 | 46 | 0.62 [0.39, 0.99] | 0.0435 | 0.1784 |
| 6m | Visual field defects | 25149 | 25126 | 10 | 26 | 0.37 [0.18, 0.78] | 0.006 | 0.4078 |
| 12m | Visual field defects | 25149 | 25126 | 17 | 45 | 0.37 [0.21, 0.64] | 0.0002 | 0.8396 |
| 24m | Visual field defects | 25103 | 25062 | 35 | 68 | 0.5 [0.33, 0.75] | 0.0007 | 0.2636 |
| 36m | Visual field defects | 23782 | 23765 | 43 | 68 | 0.62 [0.42, 0.9] | 0.0119 | 0.3165 |
| 48m | Visual field defects | 22491 | 22453 | 56 | 82 | 0.66 [0.47, 0.93] | 0.0154 | 0.0385 |
| 60m | Visual field defects | 20792 | 20751 | 56 | 83 | 0.65 [0.46, 0.92] | 0.0129 | 0.6049 |

| | | | | | | | | |
|---|---|---|---|---|---|---|---|---|
| 120m | Visual field defects | 22491 | 22453 | 73 | 120 | 0.61 [0.45, 0.81] | 0.0007 | 0.8722 |
| 6m | Subjective visual disturbances | 25278 | 25278 | 27 | 67 | 0.39 [0.25, 0.61] | <0.0001 | 0.6444 |
| 12m | Subjective visual disturbances | 25278 | 25278 | 42 | 99 | 0.41 [0.29, 0.59] | <0.0001 | 0.6289 |
| 24m | Subjective visual disturbances | 25239 | 25239 | 73 | 143 | 0.5 [0.38, 0.66] | <0.0001 | 0.0721 |
| 36m | Subjective visual disturbances | 23900 | 23900 | 86 | 161 | 0.52 [0.4, 0.68] | <0.0001 | 0.1604 |
| 48m | Subjective visual disturbances | 22617 | 22617 | 107 | 189 | 0.55 [0.43, 0.69] | <0.0001 | 0.0408 |
| 60m | Subjective visual disturbances | 20905 | 20905 | 103 | 176 | 0.57 [0.44, 0.72] | <0.0001 | 0.1011 |
| 120m | Subjective visual disturbances | 22617 | 22617 | 139 | 247 | 0.56 [0.45, 0.69] | <0.0001 | 0.0848 |
| 6m | Conjunctival hyperemia | 25269 | 25268 | 0 | 10 | nan [nan, nan] | 0.3109 | nan |
| 12m | Conjunctival hyperemia | 25269 | 25268 | 0 | 10 | nan [nan, nan] | 0.078 | nan |
| 24m | Conjunctival hyperemia | 25227 | 25227 | 0 | 10 | nan [nan, nan] | 0.0424 | nan |
| 36m | Conjunctival hyperemia | 23892 | 23888 | 10 | 10 | 0.65 [0.11, 3.89] | 0.6334 | 0.0526 |
| 48m | Conjunctival hyperemia | 22607 | 22611 | 10 | 10 | 0.24 [0.03, 2.16] | 0.1675 | 0.514 |
| 60m | Conjunctival hyperemia | 20898 | 20894 | 10 | 10 | 0.24 [0.03, 2.18] | 0.171 | 0.7892 |
| 120m | Conjunctival hyperemia | 22607 | 22611 | 10 | 10 | 0.5 [0.09, 2.72] | 0.411 | 0.1033 |
| 6m | Color vision deficiencies | 25273 | 25260 | 10 | 10 | 0.65 [0.11, 3.9] | 0.6373 | 0.0652 |

| | | | | | | | | |
|---|---|---|---|---|---|---|---|---|
| 12m | Color vision deficiencies | 25273 | 25260 | 10 | 10 | 0.98 [0.2, 4.83] | 0.9761 | 0.567 |
| 24m | Color vision deficiencies | 25232 | 25221 | 10 | 10 | 0.78 [0.21, 2.91] | 0.7121 | 0.324 |
| 36m | Color vision deficiencies | 23893 | 23883 | 10 | 10 | 0.58 [0.14, 2.45] | 0.4571 | 0.1578 |
| 48m | Color vision deficiencies | 22615 | 22601 | 10 | 10 | 0.64 [0.11, 3.86] | 0.6278 | 0.1919 |
| 60m | Color vision deficiencies | 20903 | 20892 | 10 | 10 | 0.96 [0.19, 4.78] | 0.9646 | 0.7898 |
| 120m | Color vision deficiencies | 22615 | 22601 | 10 | 10 | 1.0 [0.2, 4.96] | 0.9991 | 0.9034 |
| 6m | Inguinal Hernia | 24958 | 24958 | 25 | 28 | 0.87 [0.51, 1.5] | 0.6215 | 0.9353 |
| 12m | Inguinal Hernia | 24958 | 24958 | 48 | 44 | 1.06 [0.7, 1.59] | 0.782 | 0.1916 |
| 24m | Inguinal Hernia | 24920 | 24909 | 98 | 93 | 1.03 [0.77, 1.37] | 0.8493 | 0.741 |
| 36m | Inguinal Hernia | 23607 | 23611 | 119 | 103 | 1.13 [0.87, 1.47] | 0.3713 | 0.2241 |
| 48m | Inguinal Hernia | 22342 | 22321 | 142 | 122 | 1.13 [0.88, 1.43] | 0.3365 | 0.4522 |
| 60m | Inguinal Hernia | 20653 | 20620 | 157 | 132 | 1.15 [0.92, 1.46] | 0.2222 | 0.7942 |
| 120m | Inguinal Hernia | 22342 | 22321 | 202 | 180 | 1.13 [0.92, 1.38] | 0.2391 | 0.7852 |
