# Supplementary material for: Biologic therapy is associated with reduced ocular disease in psoriasis: a real-world study
Source: Eye (Lond). 2026 Feb 5;40(5):676–81. doi: 10.1038/s41433-026-04274-x (PMC13013609; doi:10.1038/s41433-026-04274-x)
Supplement: Supplementary file 11 — Supplementary Table S10 [file 41433_2026_4274_MOESM11_ESM.pdf]

Supplementary Table S10: Characteristics of patients with psoriasis treated with biologic agents compared with those treated with systemic non-biologic therapies in the US and EMEA networks.

**Characteristic Name**	**Network**	**Before PSM**				**After PSM**			
		**Biological (n=82,521)**	**Systemic (n=67,670)**	***P***	**Std diff.**	**Biological (n=56,017)**	**Systemic (n=56,017)**	***P***	**Std diff.**
Age at Index (mean±SD)	US	47.85±16.37	55.81±17.03	**<0.0001**	**0.48**	52.49±16.09	52.7±16.45	0.0294	0.01
White (%)		65089 (79.08)	52305 (78.19)	<0.0001	0.02	43805 (78.2)	44148 (78.81)	0.0126	0.01
Female (%)		44181 (53.68)	41096 (61.43)	**<0.0001**	**0.16**	32807 (58.57)	32429 (57.89)	0.0220	0.01
Hypertensive diseases (%)		17394 (21.13)	20717 (30.97)	**<0.0001**	**0.23**	14906 (26.61)	14507 (25.9)	0.0067	0.02
Hyperlipidemia, unspecified (%)		8957 (10.88)	11793 (17.63)	**<0.0001**	**0.19**	7897 (14.1)	7618 (13.6)	0.0158	0.01
Diabetes mellitus (%)		8440 (10.25)	9186 (13.73)	**<0.0001**	**0.11**	6999 (12.49)	6838 (12.21)	0.1438	0.01
Nicotine dependence (%)		4449 (5.4)	4154 (6.21)	<0.0001	0.03	3435 (6.13)	3361 (6.0)	0.3544	0.01
Long term (current) use of systemic steroids (%)		1047 (1.27)	1555 (2.33)	<0.0001	0.08	899 (1.6)	880 (1.57)	0.6498	0.00
Family history of other specified eye disorder (%)		29 (0.04)	32 (0.05)	0.2311	0.01	20 (0.04)	22 (0.04)	0.7576	0.00
**Characteristic Name**	**Network**	**Before PSM**				**After PSM**			
		**Biological (n=3,408)**	**Systemic (n=3,333)**	***P***	**Std diff.**	**Biological (n=2,782)**	**Systemic (n=2,782)**	***P***	**Std diff.**
Age at Index (mean±SD)	EMEA	46.54+16.24	53.09+16.79	**<0.0001**	**0.40**	49.41+15.25	49.69_15.49	0.5001	0.02
White (%)		599 (17.58)	696 (20.88)	0.0006	0.08	513 (18.44)	511 (18.37)	0.9448	0.00
Female (%)		1555 (45.63)	1440 (43.2)	0.0453	0.05	1164 (41.84)	1217 (43.75)	0.1510	0.04
Hypertensive diseases (%)		433 (12.7)	564 (16.92)	**<0.0001**	**0.12**	372 (13.37)	401 (14.41)	0.2610	0.03
Hyperlipidemia, unspecified (%)		120 (3.52)	234 (7.02)	**<0.0001**	**0.16**	116 (4.17)	110 (3.95)	0.6837	0.01
Diabetes mellitus (%)		243 (7.13)	325 (9.75)	0.0001	0.09	200 (7.19)	219 (7.87)	0.3344	0.03
Nicotine dependence (%)		148 (4.34)	151 (4.53)	0.7082	0.01	108 (3.88)	125 (4.49)	0.2552	0.03
Long term (current) use of systemic steroids (%)		10 (0.29)	14 (0.42)	0.3829	0.02	10 (0.36)	10 (0.36)	1	0.00
Family history of other specified eye disorder (%)		0 (0.0)	0 (0.0)	NA	NA	0 (0.0)	0 (0.0)	NA	NA
