# Supplementary material for: Biologic therapy is associated with reduced ocular disease in psoriasis: a real-world study
Source: Eye (Lond). 2026 Feb 5;40(5):676–81. doi: 10.1038/s41433-026-04274-x (PMC13013609; doi:10.1038/s41433-026-04274-x)
Supplement: Supplementary file 12 — Supplementary Table S11 [file 41433_2026_4274_MOESM12_ESM.pdf]

Supplementary Table S11: Summary of ocular outcomes across the US and EMEA networks in patients with confirmed diagnosis of psoriasis who were prescribed with biologic agents vs. patients with a confirmed diagnosis of psoriasis who were prescribed with non-biologic systemic therapy. Over a follow-up period of 5 years across the US network, biologic therapy was associated with a lower HR for most ophthalmic outcomes compared with systemic non-biologic treatments.

Outcome	Network	Patients in cohort		Patients with outcome		Survival probability at the end of time window		HR [95% CI]	Log-rank test p-value	Proportionality test p-value
		Biological	Systemic	Biological	Systemic	Biological	Systemic			
**Blepharitis**	**US**	**55297**	**54671**	**427**	**588**	**0.9865**	**0.9826**	**0.75 [0.66, 0.85]**	**<0.0001**	**0.3381**
**Conjunctivitis**	**US**	**53857**	**52981**	**1081**	**1247**	**0.9649**	**0.9609**	**0.9 [0.83, 0.97]**	**0.0092**	**0.8963**
**Keratitis**	**US**	**55452**	**54573**	**333**	**616**	**0.9893**	**0.9819**	**0.56 [0.49, 0.64]**	**<0.0001**	**0.0579**
**Dry eye syndrome**	**US**	**54310**	**52283**	**1102**	**1626**	**0.9646**	**0.9498**	**0.68 [0.63, 0.73]**	**<0.0001**	**0.0252**
**Iridocyclitis**	**US**	**55265**	**55422**	**228**	**199**	**0.9930**	**0.9941**	**1.21 [1.0, 1.47]**	**0.0475**	**0.4277**
**Glaucoma**	**US**	**54459**	**53959**	**705**	**877**	**0.9776**	**0.9734**	**0.84 [0.76, 0.92]**	**0.0004**	**0.8934**
**Age-related cataract**	**US**	**53626**	**52651**	**1543**	**1859**	**0.9497**	**0.9411**	**0.85 [0.8, 0.91]**	**<0.0001**	**0.6036**
**Age-related macular degeneration**	**US**	**55227**	**54889**	**508**	**697**	**0.9830**	**0.9783**	**0.77 [0.68, 0.86]**	**<0.0001**	**0.3957**
Retinal vascular occlusions	**US**	55883	55837	93	90	0.9970	0.9973	1.1 [0.82, 1.46]	0.5355	0.6086
Blepharitis	**EMEA**	2757	2768	21	14	0.9869	0.9901	1.04 [0.53, 2.05]	0.911	0.122
**Conjunctivitis**	**EMEA**	**2719**	**2689**	**56**	**71**	**0.9679**	**0.9455**	**0.55 [0.39, 0.78]**	**0.0007**	**0.3985**
Keratitis	**EMEA**	2759	2752	20	20	0.9884	0.9821	0.7 [0.37, 1.3]	0.2541	0.9917
**Dry eye syndrome**	**EMEA**	**2718**	**2680**	**57**	**64**	**0.9701**	**0.9531**	**0.64 [0.45, 0.91]**	**0.0127**	**0.8712**
Iridocyclitis	**EMEA**	NA	NA	NA	NA	NA	NA	NA	NA	NA
Glaucoma	**EMEA**	2733	2729	23	19	0.9879	0.9844	0.88 [0.48, 1.61]	0.674	0.5493
Age-related cataract	**EMEA**	2751	2758	22	22	0.9875	0.9769	0.69 [0.38, 1.25]	0.2238	0.1028
Age-related macular degeneration	**EMEA**	2765	2762	14	17	0.9922	0.9871	0.59 [0.29, 1.21]	0.1445	0.701
Retinal vascular occlusions	**EMEA**	NA	NA	NA	NA	NA	NA	NA	NA	NA
