# Supplementary material for: Biologic therapy is associated with reduced ocular disease in psoriasis: a real-world study
Source: Eye (Lond). 2026 Feb 5;40(5):676–81. doi: 10.1038/s41433-026-04274-x (PMC13013609; doi:10.1038/s41433-026-04274-x)
Supplement: Supplementary file 13 — Supplementary Table S12 [file 41433_2026_4274_MOESM13_ESM.pdf]

Supplementary Table S12: Stratified outcomes with different pathological mechanisms. Patients were grouped based on keratitis subcodes to account for potentially distinct pathophysiological mechanisms. Over a follow-up period of 5 years, several conditions demonstrated statistically significant associations with the exposure.

**Outcome**	**Patients in cohort**		**Patients with outcome**		**Survival probability at the end of time window**		**HR [95% CI]**	**Log-rank test p-value**	**Proportionality test p-value**
	**Biological**	**Systemic**	**Biological**	**Systemic**	**Biological**	**Systemic**			
**Corneal ulcer**	**62361**	**62295**	**52**	**92**	**0.99856**	**0.99739**	**0.58 [0.41, 0.81]**	**0.0013**	**0.4858**
Viral keratitis	62450	62403	39	51	0.99898	0.99855	0.78 [0.51, 1.18]	0.2347	0.2312
**Other and unspecified superficial keratitis without conjunctivitis**	**62319**	**62066**	**94**	**212**	**0.99724**	**0.99437**	**0.45 [0.35, 0.57]**	**<0.0001**	**0.0515**
**Keratoconjunctivitis**	**62252**	**61577**	**196**	**431**	**0.9943**	**0.98839**	**0.46 [0.38, 0.54]**	**<0.0001**	**0.0369**
Interstitial and deep keratitis	NA	NA	NA	NA	NA	NA	NA	NA	NA
**Corneal neovascularization**	**62460**	**62424**	**13**	**30**	**0.99964**	**0.99915**	**0.44 [0.23, 0.85]**	**0.0115**	**0.715**
Other keratitis	62452	62421	34	43	0.99902	0.9989	0.8 [0.51, 1.26]	0.3338	0.1749
