# Supplementary material for: Biologic therapy is associated with reduced ocular disease in psoriasis: a real-world study
Source: Eye (Lond). 2026 Feb 5;40(5):676–81. doi: 10.1038/s41433-026-04274-x (PMC13013609; doi:10.1038/s41433-026-04274-x)
Supplement: Supplementary file 14 — Supplementary Table S13 [file 41433_2026_4274_MOESM14_ESM.pdf]

Supplementary Table S13: Baseline characteristics of patients stratified according to therapeutic class. Three groups were defined: patients with psoriasis prescribed TNF inhibitors, IL-23 inhibitors, or IL-17 inhibitors.

Characteristic Name	Before PSM				After PSM			
	**IL-23 inhibitors (n=13,627)**	**TNF inhibitors (n=34,617)**	***P***	**Std diff.**	**IL-23 inhibitors (n=13,615)**	**TNF inhibitors (n=13,615)**	***P***	**Std diff.**
Age at Index (mean±SD)	49.86±15.82	47.93±16.53	**<0.0001**	**0.12**	49.84±15.81	49.88±15.74	0.8267	0.00
White (%)	10199 (74.84)	26610 (77.28)	<0.0001	0.06	10199 (74.91)	10194 (74.87)	0.9443	0.00
Female (%)	7094 (52.06)	18181 (52.8)	0.1400	0.01	7088 (52.06)	7112 (52.24)	0.7709	0.00
Hypertensive diseases (%)	3150 (23.12)	6964 (20.23)	<0.0001	0.07	3138 (23.05)	3115 (22.88)	0.7404	0.00
Hyperlipidemia, unspecified (%)	1668 (12.24)	3451 (10.02)	<0.0001	0.07	1656 (12.16)	1595 (11.72)	0.2543	0.01
Diabetes mellitus (%)	1549 (11.37)	3247 (9.43)	<0.0001	0.06	1537 (11.29)	1525 (11.2)	0.8179	0.00
Nicotine dependence (%)	684 (5.02)	1826 (5.3)	0.2074	0.01	684 (5.02)	620 (4.55)	0.0693	0.02
Long term (current) use of systemic steroids (%)	94 (0.69)	543 (1.58)	<0.0001	0.08	94 (0.69)	94 (0.69)	1.0000	0.00
Family history of other specified eye disorder (%)	10 (0.07)	11 (0.03)	0.0501	0.02	10 (0.07)	10 (0.07)	1.0000	0.00

Characteristic Name	Before PSM				After PSM			
	**IL-17 inhibitors (n=11,384)**	**TNF inhibitors (n=34,617)**	***P***	**Std diff.**	**IL-17 inhibitors (n=11,384)**	**TNF inhibitors (n=11,384))**	***P***	**Std diff.**
Age at Index (mean±SD)	49.38±15.9	47.93±16.53	<0.0001	0.09	49.38±15.9	49.36±15.95	0.9363	0.00
White (%)	7973 (70.04)	26610 (77.28)	**<0.0001**	**0.17**	7973 (70.04)	7988 (70.17)	0.8281	0.00
Female (%)	6189 (54.37)	18181 (52.8)	0.0038	0.03	6189 (54.37)	6240 (54.81)	0.4972	0.01
Hypertensive diseases (%)	2441 (21.44)	6964 (20.23)	0.0053	0.03	2441 (21.44)	2402 (21.1)	0.5277	0.01
Hyperlipidemia, unspecified (%)	1274 (11.19)	3247 (9.43)	<0.0001	0.06	1274 (11.19)	1224 (10.75)	0.2890	0.01
Diabetes mellitus (%)	1212 (10.65)	3451 (10.02)	0.0564	0.02	1212 (10.65)	1108 (9.73)	0.0227	0.03
Nicotine dependence (%)	597 (5.24)	1826 (5.3)	0.8069	0.00	597 (5.24)	539 (4.74)	0.0775	0.02
Long term (current) use of systemic steroids (%)	122 (1.07)	543 (1.58)	<0.0001	0.04	122 (1.07)	98 (0.86)	0.1040	0.02
Family history of other specified eye disorder (%)	10 (0.09)	11 (0.03)	0.0157	0.02	10 (0.09)	0 (0.0)	0.0016	0.04

Characteristic Name	Before PSM				After PSM			
	**IL-17 inhibitors (n=11,384)**	**IL-23 inhibitors (n=13,627)**	***P***	**Std diff.**	**IL-17 inhibitors (n=11,226)**	**IL-23 inhibitors (n=11,226)**	***P***	**Std diff.**
Age at Index (mean±SD)	49.38±15.9	49.86±15.82	0.0158	0.03	49.44±15.89	49.92±15.56	0.0227	0.03
White (%)	7973 (70.04)	10199 (74.84)	**<0.0001**	**0.11**	7959 (70.9)	7927 (70.61)	0.6387	0.01
Female (%)	6189 (54.37)	7094 (52.06)	0.0003	0.05	6045 (53.85)	6056 (53.95)	0.8829	0.00
Hypertensive diseases (%)	2441 (21.44)	3150 (23.12)	0.0016	0.04	2424 (21.59)	2461 (21.92)	0.5495	0.01
Hyperlipidemia, unspecified (%)	1274 (11.19)	1549 (11.37)	0.6614	0.01	1258 (11.21)	1220 (10.87)	0.4183	0.01
Diabetes mellitus (%)	1212 (10.65)	1668 (12.24)	<0.0001	0.05	1208 (10.76)	1198 (10.67)	0.8292	0.00
Nicotine dependence (%)	597 (5.24)	684 (5.02)	0.4220	0.01	584 (5.2)	551 (4.91)	0.3148	0.01
Long term (current) use of systemic steroids (%)	122 (1.07)	94 (0.69)	0.0012	0.04	89 (0.79)	88 (0.78)	0.9398	0.00
Family history of other specified eye disorder (%)	10 (0.09)	10 (0.07)	0.6871	0.01	10 (0.09)	0 (0.0)	0.0016	0.04
