# Supplementary material for: Biologic therapy is associated with reduced ocular disease in psoriasis: a real-world study
Source: Eye (Lond). 2026 Feb 5;40(5):676–81. doi: 10.1038/s41433-026-04274-x (PMC13013609; doi:10.1038/s41433-026-04274-x)
Supplement: Supplementary file 15 — Supplementary Table S14 [file 41433_2026_4274_MOESM15_ESM.pdf]

Supplementary Table S14: Ocular outcomes in patients stratified according to therapeutic class.

Outcome	Patients in cohort		Patients with outcome		Survival probability at the end of time window		HR [95% CI]	Log-rank test p-value	Proportionality test p-value
	IL-23 inhibitors	TNF inhibitors	IL-23 inhibitors	TNF inhibitors	IL-23 inhibitors	TNF inhibitors			
Blepharitis	13434	13477	61	111	0.9878	0.9870	0.85 [0.61, 1.17]	0.3061	0.262
**Conjunctivitis**	**13063**	**13145**	**185**	**253**	**0.9538**	**0.9678**	**1.26 [1.03, 1.53]**	**0.0215**	**0.1844**
Keratitis	13480	13499	45	64	0.9919	0.9922	1.12 [0.76, 1.66]	0.5581	0.7892
Dry eye syndrome	13252	13252	174	259	0.9635	0.9695	1.06 [0.87, 1.29]	0.584	0.0106
**Iridocyclitis**	**13547**	**13323**	**14**	**68**	**0.9980**	**0.9915**	**0.32 [0.18, 0.58]**	**0.0001**	**0.6341**
Glaucoma	13299	13265	115	158	0.9765	0.9804	1.2 [0.94, 1.54]	0.1425	0.2839
Age-related cataract	13085	13144	200	329	0.9531	0.9584	1.03 [0.86, 1.23]	0.7704	0.0085
Age-related macular degeneration	13461	13430	77	115	0.9778	0.9846	1.27 [0.94, 1.71]	0.1218	0.1919
Retinal vascular occlusions	13581	13578	10	21	0.9984	0.9974	0.79 [0.37, 1.7]	0.5435	0.4711
**Outcome**	**Patients in cohort**		**Patients with outcome**		**Survival probability at the end of time window**		**HR [95% CI]**	**Log-rank test p-value**	**Proportionality test p-value**
	**IL-17 inhibitors**	**TNF inhibitors**	**IL-17 inhibitors**	**TNF inhibitors**	**IL-17 inhibitors**	**TNF inhibitors**			
Blepharitis	11290	11279	67	84	0.9870	0.9879	1.03 [0.75, 1.43]	0.8441	0.3986
**Conjunctivitis**	**10987**	**10970**	**207**	**225**	**0.9596**	**0.9655**	**1.21 [1.0, 1.46]**	**0.049**	**0.4337**
Keratitis	11283	11287	55	61	0.9885	0.9907	1.21 [0.84, 1.75]	0.3098	0.5733
Dry eye syndrome	11089	11085	198	213	0.9614	0.9693	1.2 [0.99, 1.46]	0.0668	0.2586
Iridocyclitis	11311	11132	37	55	0.9935	0.9916	0.86 [0.56, 1.31]	0.4786	0.5591
Glaucoma	11153	11093	82	137	0.9847	0.9794	0.78 [0.59, 1.02]	0.0727	0.6366
Age-related cataract	11101	10999	184	267	0.9652	0.9594	0.89 [0.74, 1.08]	0.2357	0.868

Age-related macular degeneration	11277	11245	48	93	0.9885	0.9849	0.73 [0.51, 1.04]	0.0793	0.771
Retinal vascular occlusions	11365	11352	12	17	0.9974	0.9974	0.96 [0.46, 2.02]	0.9117	0.9101

**Outcome**	**Patients in cohort**		**Patients with outcome**		**Survival probability at the end of time window**		**HR [95% CI]**	**Log-rank test p-value**	**Proportionality test p-value**
	**IL-17 inhibitors**	**IL-23 inhibitors**	**IL-17 inhibitors**	**IL-23 inhibitors**	**IL-17 inhibitors**	**IL-23 inhibitors**			
Blepharitis	11132	11073	66	53	0.9870	0.9872	1.0 [0.69, 1.44]	0.9946	0.6596
Conjunctivitis	10836	10778	206	150	0.9592	0.9521	1.08 [0.87, 1.34]	0.4743	0.005
Keratitis	11126	11113	54	40	0.9885	0.9915	1.08 [0.71, 1.63]	0.7256	0.3191
Dry eye syndrome	10935	10916	195	151	0.9616	0.9613	1.04 [0.84, 1.28]	0.7489	0.1724
**Iridocyclitis**	**11153**	**11170**	**35**	**12**	**0.9936**	**0.9979**	**2.45 [1.27, 4.74]**	**0.0058**	**0.964**
**Glaucoma**	**11000**	**10961**	**79**	**98**	**0.9852**	**0.9759**	**0.64 [0.48, 0.87]**	**0.0034**	**0.3137**
Age-related cataract	10943	10789	182	166	0.9650	0.9519	0.86 [0.69, 1.06]	0.1492	0.0243
**Age-related macular degeneration**	**11120**	**11096**	**48**	**68**	**0.9884**	**0.9764**	**0.51 [0.35, 0.75]**	**0.0004**	**0.7757**
Retinal vascular occlusions	11207	11199	12	10	0.9974	0.9981	0.96 [0.41, 2.24]	0.9253	0.6011
