# Supplementary material for: Biologic therapy is associated with reduced ocular disease in psoriasis: a real-world study
Source: Eye (Lond). 2026 Feb 5;40(5):676–81. doi: 10.1038/s41433-026-04274-x (PMC13013609; doi:10.1038/s41433-026-04274-x)
Supplement: Supplementary file 16 — Supplementary Table S15 [file 41433_2026_4274_MOESM16_ESM.pdf]

Supplementary Table S15: Patient characteristics before and after propensity score matching for patients with confirmed diagnosis of arthropathic psoriasis who were prescribed with biologic agents vs. patients with a confirmed diagnosis of arthropathic psoriasis who were prescribed with non-biologic systemic therapy for a follow-up period of 60 months. Std. diff, standard difference; SD, standard deviation.

**Characteristic Name**	**Before PSM**				**After PSM**			
	**Biological (n=40,831)**	**Systemic (n=29,749)**	***P***	**Std diff.**	**Biological (n=26,882)**	**Systemic (n=26,882)**	***P***	**Std diff.**
Age at Index (mean±SD)	50.42±14.75	56.28±15.43	**<0.0001**	**0.39**	54.36±14.53	54.83±15.12	0.0002	0.03
White (%)	32375 (79.55)	21739 (73.86)	**<0.0001**	**0.13**	19695 (73.26)	20388 (75.84)	<0.0001	0.06
Female (%)	22646 (55.64)	18036 (61.28)	**<0.0001**	**0.11**	16226 (60.36)	16063 (59.75)	0.1512	0.01
Hypertensive diseases (%)	9535 (23.43)	8755 (29.74)	**<0.0001**	**0.14**	7509 (27.93)	7421 (27.61)	0.3968	0.01
Hyperlipidemia, unspecified (%)	4814 (11.83)	4870 (16.55)	**<0.0001**	**0.14**	3907 (14.53)	3967 (14.76)	0.4642	0.01
Diabetes mellitus (%)	4579 (11.25)	3964 (13.47)	<0.0001	0.07	3484 (12.96)	3478 (12.94)	0.9386	0.00
Nicotine dependence (%)	2179 (5.35)	1586 (5.39)	0.8420	0.00	1484 (5.52)	1482 (5.51)	0.9699	0.00
Long term (current) use of systemic steroids (%)	592 (1.46)	693 (2.35)	<0.0001	0.07	483 (1.8)	501 (1.86)	0.5625	0.00
Family history of other specified eye disorder (%)	13 (0.03)	10 (0.03)	0.8834	0.00	10 (0.04)	10 (0.04)	1.0000	0.00
