# Supplementary material for: Biologic therapy is associated with reduced ocular disease in psoriasis: a real-world study
Source: Eye (Lond). 2026 Feb 5;40(5):676–81. doi: 10.1038/s41433-026-04274-x (PMC13013609; doi:10.1038/s41433-026-04274-x)
Supplement: Supplementary file 17 — Supplementary Table S16 [file 41433_2026_4274_MOESM17_ESM.pdf]

Supplementary Table S16: Summary of hazard ratios (HRs) with 95% confidence interval (95% CI), log-rank tests p-values and proportionality tests p-values for ocular outcomes in patients with confirmed diagnosis of arthropathic psoriasis who were prescribed with biologic agents vs. patients with a confirmed diagnosis of arthropathic psoriasis who were prescribed with non-biologic systemic therapy.

**Outcome**	**Patients in cohort**		**Patients with outcome**		**Survival probability at the end of time window**		**HR [95% CI]**	**Log-rank test p-value**	**Proportionality test p-value**
	**Biological**	**Systemic**	**Biological**	**Systemic**	**Biological**	**Systemic**			
Blepharitis	26575	26413	176	195	0.9886	0.9877	0.91 [0.74, 1.11]	0.3427	0.5358
Conjunctivitis	25923	25761	475	518	0.9684	0.9650	0.92 [0.82, 1.05]	0.2108	0.2414
**Keratitis**	**26601**	**26433**	**148**	**211**	**0.9903**	**0.9872**	**0.7 [0.57, 0.87]**	**0.0009**	**0.0511**
**Dry eye syndrome**	**25962**	**25503**	**584**	**696**	**0.9616**	**0.9545**	**0.83 [0.74, 0.92]**	**0.0008**	**0.3567**
Iridocyclitis	26470	26553	123	103	0.9922	0.9941	1.21 [0.93, 1.57]	0.1566	0.0507
**Glaucoma**	**26101**	**25999**	**336**	**391**	**0.9783**	**0.9747**	**0.86 [0.75, 1.0]**	**0.0499**	**0.6112**
**Age-related cataract**	**25686**	**25520**	**725**	**825**	**0.9520**	**0.9452**	**0.88 [0.8, 0.97]**	**0.011**	**0.6523**
**Age-related macular degeneration**	**26499**	**26359**	**237**	**334**	**0.9841**	**0.9777**	**0.72 [0.61, 0.84]**	**0.0001**	**0.6742**
Retinal vascular occlusions	26811	26795	44	37	0.9971	0.9977	1.21 [0.78, 1.87]	0.3965	0.6835
