# Supplementary material for: Biologic therapy is associated with reduced ocular disease in psoriasis: a real-world study
Source: Eye (Lond). 2026 Feb 5;40(5):676–81. doi: 10.1038/s41433-026-04274-x (PMC13013609; doi:10.1038/s41433-026-04274-x)
Supplement: Supplementary file 18 — Supplementary Table S17 [file 41433_2026_4274_MOESM18_ESM.pdf]

Supplementary Table S17: Patient characteristics before and after propensity score matching using an extended list of covariates for patients with confirmed diagnosis of psoriasis who were prescribed with biologic agents vs. patients with a confirmed diagnosis of psoriasis who were prescribed with non-biologic systemic therapy for a follow-up period of 60 months. Std. diff, standard difference; SD, standard deviation.

**Characteristic Name**	**Before PSM**				**After PSM**			
	**Biological (n=89,088)**	**Systemic (n=81,568)**	***P***	**Std diff.**	**Biological (n=62,652)**	**Systemic (n=62,652)**	***P***	**Std diff.**
Age at Index (mean±SD)	47.91±16.34	55.18±17.12	**<0.0001**	**0.43**	51.48±15.87	51.76±16.83	0.0020	0.02
White (%)	67715 (76.19)	55060 (68.21)	**<0.0001**	**0.18**	44346 (70.78)	45009 (71.84)	<0.0001	0.02
Female (%)	47468 (53.41)	47040 (58.27)	**<0.0001**	**0.10**	35310 (56.36)	34837 (55.6)	0.0071	0.02
Factors influencing health status and contact with health services (%)	42839 (48.2)	40458 (50.12)	<0.0001	0.04	29997 (47.88)	30003 (47.89)	0.9729	0.00
Hypertensive diseases (%)	18646 (20.98)	23228 (28.78)	**<0.0001**	**0.18**	15246 (24.33)	15106 (24.11)	0.3559	0.01
Hyperlipidemia, unspecified (%)	9467 (10.65)	12924 (16.01)	**<0.0001**	**0.16**	7995 (12.76)	7912 (12.63)	0.4812	0.00
Diabetes mellitus (%)	9085 (10.22)	10613 (13.15)	<0.0001	0.09	7246 (11.56)	7288 (11.63)	0.7110	0.00
Nicotine dependence (%)	4805 (5.41)	4540 (5.62)	0.0496	0.01	3405 (5.44)	3461 (5.52)	0.4870	0.00
Atopic dermatitis (%)	910 (1.02)	1908 (2.36)	**<0.0001**	**0.10**	801 (1.28)	809 (1.29)	0.8410	0.00
Rosacea (%)	760 (0.86)	962 (1.19)	<0.0001	0.03	575 (0.92)	598 (0.95)	0.4999	0.00
Ankylosing spondylitis (%)	1872 (2.11)	531 (0.66)	**<0.0001**	**0.12**	548 (0.88)	518 (0.83)	0.3561	0.01
Arthropathic psoriasis (%)	21548 (24.24)	14966 (18.54)	**<0.0001**	**0.14**	13491 (21.53)	13469 (21.5)	0.8798	0.00
Crohn's disease (%)	4766 (5.36)	500 (0.62)	**<0.0001**	**0.28**	531 (0.85)	499 (0.8)	0.3167	0.01
Ulcerative colitis (%)	2047 (2.3)	375 (0.46)	**<0.0001**	**0.16**	411 (0.66)	369 (0.59)	0.1314	0.01
Rheumatoid arthritis with rheumatoid factor (%)	1273 (1.43)	1635 (2.02)	<0.0001	0.05	1062 (1.7)	1117 (1.78)	0.2346	0.01
Other rheumatoid arthritis (%)	5044 (5.68)	6272 (7.77)	<0.0001	0.08	4036 (6.44)	4163 (6.64)	0.1468	0.01
Family history of other specified eye disorder (%)	30 (0.03)	33 (0.04)	0.4469	0.00	21 (0.03)	17 (0.03)	0.5163	0.00
Corticosteroids for systemic use (%)	35571 (40.02)	37235 (46.13)	**<0.0001**	**0.12**	26513 (42.32)	25977 (41.46)	0.0021	0.02
Age-related cataract (%)	1196 (1.35)	2412 (2.99)	**<0.0001**	**0.11**	1092 (1.74)	1074 (1.71)	0.6964	0.00
Dry eye syndrome (%)	904 (1.02)	2813 (3.48)	**<0.0001**	**0.17**	832 (1.33)	853 (1.36)	0.6065	0.00
Glaucoma (%)	952 (1.07)	1855 (2.3)	<0.0001	0.10	821 (1.31)	849 (1.36)	0.4903	0.00

Iridocyclitis (%)	613 (0.69)	425 (0.53)	<0.0001	0.02	310 (0.49)	329 (0.52)	0.4511	0.00
Chorioretinal inflammation (%)	83 (0.09)	63 (0.08)	0.2819	0.01	50 (0.08)	54 (0.09)	0.6948	0.00
Panuveitis (%)	55 (0.06)	37 (0.05)	0.1563	0.01	30 (0.05)	29 (0.05)	0.8964	0.00
Blepharitis (%)	258 (0.29)	631 (0.78)	<0.0001	0.07	212 (0.34)	208 (0.33)	0.8450	0.00
Acute atopic conjunctivitis (%)	220 (0.25)	363 (0.45)	<0.0001	0.03	158 (0.25)	164 (0.26)	0.7378	0.00
Presence of spectacles and contact lenses (%)	65 (0.07)	74 (0.09)	0.1828	0.01	43 (0.07)	53 (0.08)	0.3072	0.01
Long term (current) use of systemic steroids (%)	1095 (1.23)	1669 (2.07)	<0.0001	0.07	815 (1.3)	825 (1.32)	0.8037	0.00
Ophthalmological services: medical examination and evaluation, with initiation or continuation of diagnostic and treatment program; comprehensive, established patient, 1 or more visits (%)	1018 (1.14)	1892 (2.34)	<0.0001	0.09	847 (1.35)	848 (1.35)	0.9805	0.00
Ophthalmological services: medical examination and evaluation with initiation of diagnostic and treatment program; comprehensive, new patient, 1 or more visits (%)	542 (0.61)	708 (0.88)	<0.0001	0.03	412 (0.66)	426 (0.68)	0.6275	0.00
Ophthalmological services: medical examination and evaluation, with initiation or continuation of diagnostic and treatment program; intermediate, established patient (%)	401 (0.45)	847 (1.05)	<0.0001	0.07	350 (0.56)	346 (0.55)	0.8792	0.00
Ophthalmological services: medical examination and evaluation with initiation of diagnostic and treatment program; intermediate, new patient (%)	63 (0.07)	86 (0.11)	0.0133	0.01	44 (0.07)	43 (0.07)	0.9146	0.00
Visit (%)	77762 (87.49)	70889 (87.82)	0.0431	0.01	54644 (87.22)	54321 (86.7)	0.0067	0.02
Visit: Ambulatory (%)	65828 (74.07)	60730 (75.23)	<0.0001	0.03	46643 (74.45)	46004 (73.43)	<0.0001	0.02
Visit: Emergency (%)	9474 (10.66)	10567 (13.09)	<0.0001	0.08	6928 (11.06)	6848 (10.93)	0.4700	0.00
Visit: Inpatient Encounter (%)	8998 (10.12)	11031 (13.66)	**<0.0001**	**0.11**	6749 (10.77)	6638 (10.6)	0.3101	0.01
