# Supplementary material for: Biologic therapy is associated with reduced ocular disease in psoriasis: a real-world study
Source: Eye (Lond). 2026 Feb 5;40(5):676–81. doi: 10.1038/s41433-026-04274-x (PMC13013609; doi:10.1038/s41433-026-04274-x)
Supplement: Supplementary file 19 — Supplementary Table S18 [file 41433_2026_4274_MOESM19_ESM.pdf]

Supplementary Table S18: Summary of hazard ratios (HRs) with 95% confidence interval (95% CI), log-rank tests p-values and proportionality tests p-values for ocular outcomes in patients with confirmed diagnosis of psoriasis who were prescribed with biologic agents vs. patients with a confirmed diagnosis of psoriasis who were prescribed with non-biologic systemic therapy, following an extended version of potential covariates.

**Outcome**	**Patients in cohort**		**Patients with outcome**		**Survival probability at the end of time window**		**HR [95% CI]**	**Log-rank test p-value**	**Proportionality test p-value**
	**Biological**	**Systemic**	**Biological**	**Systemic**	**Biological**	**Systemic**			
**Blepharitis**	**61898**	**61650**	**441**	**511**	**0.98773**	**0.98637**	**0.88 [0.77, 1.0]**	**0.0464**	**0.3828**
**Conjunctivitis**	**60372**	**59856**	**1188**	**1356**	**0.96581**	**0.96176**	**0.89 [0.82, 0.96]**	**0.0032**	**0.9201**
**Keratitis**	**62037**	**61506**	**337**	**547**	**0.99037**	**0.9852**	**0.63 [0.55, 0.72]**	**<0.0001**	**0.1511**
**Dry eye syndrome**	**60766**	**59843**	**1168**	**1719**	**0.96681**	**0.95238**	**0.68 [0.63, 0.73]**	**<0.0001**	**0.1758**
Iridocyclitis	61988	62023	227	198	0.99387	0.99479	1.17 [0.97, 1.42]	0.097	0.9827
**Glaucoma**	**61000**	**60905**	**734**	**910**	**0.9791**	**0.97475**	**0.83 [0.75, 0.91]**	**0.0001**	**0.8094**
**Age-related cataract**	**60269**	**59981**	**1579**	**1809**	**0.95353**	**0.94783**	**0.89 [0.83, 0.95]**	**0.0007**	**0.8066**
**Age-related macular degeneration**	**61873**	**61642**	**501**	**699**	**0.98526**	**0.98017**	**0.74 [0.66, 0.83]**	**<0.0001**	**0.841**
Retinal vascular occlusions	62514	62479	93	99	0.9973	0.99727	0.97 [0.73, 1.29]	0.8347	0.4884
